# Supplementary material for: Constructing a synthetic pathway for acetyl-coenzyme A from one-carbon through enzyme design
Source: Nat Commun. 2019 Mar 26;10:1378. doi: 10.1038/s41467-019-09095-z (PMC6435721; doi:10.1038/s41467-019-09095-z)
Supplement: Supplementary file 1 — Supplementary Information [file 41467_2019_9095_MOESM1_ESM.docx]

**Constructing a Synthetic Pathway for Acetyl-Coenzyme A from One-Carbon through Enzyme Design**

Lu *et al*.

**Supplementary Note**

**Screening candidates of ThDP-dependent enzymes**

In order to design an enzyme for synthesis of glycolaldehyde from formaldehyde, we virtually screened all enzymes with cofactor ThDP in Protein Data Bank (PDB). Totally 270 protein structures with cofactor ThDP were abstracted. We firstly removed the structures with a single chain or RNA strands because ThDP-dependent enzyme usually conducts function as dimer^1^. Secondly we clustered and filtered redundant structures using cd-hit^2^ (the cutoff value of sequence identities is 95%). And then, the highest resolution structure in each cluster was retained. Finally, 37 out of 270 structures were achieved.

In order to identify an enzyme that can produce glycolaldehyde, we performed rigid docking experiments using glycolaldehyde as substrate in DOCK6^3^ program. ThDP and glycolaldehyde were parameterized in UCSF Chimera package^4^. Atom charges and hydrogen atoms were added to all proteins by Chimera using AMBER ff99SB parameters. Gasteiger-Huckel charges^5^ and AM1-BCC charges^6^ were assigned on the standard residues and non-standard residues (cofactor ThDP or substrate glycolaldehyde).

After molecular docking, 37 candidates were ranked according to the average distances between C2 atom in ThDP and two carbon atoms in glycolaldehyde. The average distance was critical for triggering the catalytic reaction. Since 2UZ1 (BAL) has been reported to produce glycolaldehyde under low concentration of formaldehyde^7^, in order to identify a better enzyme, we selected 14 proteins (including 2UZ1) from 37 candidates, which have shorter distance. By filtering impossible proteins (4TKR is not an enzyme and 2C42 is an oxidoreductase) and functional redundancy (Supplementary Table 2), we obtained six candidates (4COK, 1R9J, 3FZN, 4K9Q, 2JLC and 2UZ1). Three proteins with long distances were randomly chosen as controls (5C5I, 2C31 and 4FEG).

**Functional identification of ThDP-dependent enzymes**

The coding genes of candidate glycolaldehyde synthase were ligated into the expression vector pET-28a via *Nde*І and *Xho*I restriction sites. *E. coli* BL21(DE3) cells carrying different recombinant plasmids were inoculated into 5 mL LB (Luria Broth) medium with Kanamycine (100 μg mL^-1^) and cultured overnight at 37 °C, and then scaled up to 800 mL LB medium containing appropriate resistance. When cell OD_600_ reached 0.6, IPTG (isopropyl-β-D-thiogalactopyranoside) was added to induce gene expression with a final concentration of 0.5 mM. The cell cultures continued to grow overnight at 16 °C before being harvested by centrifugation at 6,000 g and then were resuspended in 50 mL lysis buffer (50 mM potassium phosphate buffer, pH 7.4, 5 mM MgSO_4_, 0.5 mM ThDP). The bacterial pellet was lysed by using a high-pressure homogenizer (JNBIO, China), and the cell debris was removed by centrifugation at 10,000 g for 60 min at 4 °C. The soluble protein sample was loaded onto a nickel affinity column (GE Healthcare), rinsing with 50 mL wash buffer (50 mM potassium phosphate buffer, pH 7.4, 5 mM MgSO_4_, 0.5 mM ThDP and 50 mM imidazole) and then eluting with 20 mL elution buffer (50 mM potassium phosphate buffer, pH 7.4, 5 mM MgSO_4_, 0.5 mM ThDP and 200 mM imidazole). The eluted protein was concentrated and dialyzed against lysis buffer by ultrafiltration with an Amicon Ultra centrifugal filter device (Millipore, USA) with a 30 kDa molecular-weight cutoff. The protein concentration was determined using a BCA Protein Assay Reagent Kit (Pierce, USA) with BSA as the standard.

Activity of candidate enzymes was determined with 1 mg mL^-1^ of purified enzymes in 200 µL reaction mixtures. The reaction system comprised 2 g L^-1^ formaldehyde, 1 mM ThDP, 5 mM MgSO_4_ and 50 mM phosphate buffer (pH 7.4). After incubation at 37 °C for 6 hours, the reaction was stopped by adding 200 µL of acetonitrile. Samples were detected by High Performance Liquid Chromatography (HPLC). HPLC detection conditions for glycolaldehyde: column: Aminex HPX-87H (Bio-Rad); detection wavelength: 200 nm; mobile phase: 5 mM sulphuric acid; flow rate: 0.6 mL min^-1^; sample volume: 20 μL; column temperature: 40 °C.

**Protein engineering of glycolaldehyde synthase**

The determination of glycolaldehyde is as follows: 30 μL different concentrations of glycolaldehyde were prepared, and then 150 μL spetrophotometric chromogenic reagent (1.5 g diphenylamine was dissolved into 100 mL acetic acid, then added 1.5 mL concentrated sulfuric acid) was added, keeping at 90 °C for 15 min^8^. At last, product concentration was measured by spectrophotometrically monitoring at 650 nm.

In order to obtain the desired saturation mutagenesis, oligonucleotide primers were designed with degenerate codon NNK. For 95% library coverage, the screening of 96 transformants for single-site saturation mutant was required by using NNK codon degeneracy^9^. Each single-site saturation mutant library was generated according to the PCR-based Quick Change method. PCR reaction was performed with Fastpfu DNA Polymerase (Transgen, China) under the following conditions: the reaction was started at 94 °C (5 min), followed by 30 cycles 94 °C (20 s), 58 °C (20 s), 72 °C (3.5 min), with a ﬁnal extension at 72 °C (5 min). The PCR product was digested with *Dpn*I restriction enzyme and transformed into *E. coli* BL21 (DE3) competent cells to create the library for screening.

Each of the mutant colonies was picked and incubated 24 hours in 200 μL LB medium with 100 μg mL^-1^ kanamycin while shaking at 37 °C in 96-well microplate, and then scaled up to 1 mL LB medium for [protein expression](D:/Users/SunTao/AppData/Local/Youdao/Dict/7.5.2.0/resultui/dict/javascript:;) as well as BFD. The cell pellets were harvested by centrifugation at 3,300 g for 1 min and lysed by the re-suspension in 150 μL lysis buffer with 1 U DNase I and 1 mg mL^-1^ lysozyme, followed by 1 hour at 37 °C. Subsequently, 150 μL lysis buffer with 2 g L^-1^ formaldehyde was added directly to the crude lysates for condensation assay and the plates were further incubated at 37 °C at 750 rpm for 90 min. After removing cells by centrifugation, 30 μL of the samples was used for a coloration assay.

To further manipulate the binding pocket of BFD, we utilized the structure-based combinatorial active-site saturation test (CASTing) in combination with iterative saturation mutagenesis (ISM) as a method for manipulating^9^. In each cycle, the best variant was fixed into the population by retention in the next round parental enzyme. About 3,072 transformants would need to be screened for 95% library coverage at each combinatorial double-sites saturation mutant library. The cloning and screening methods for ISM were executed as well as single point saturation mutation.

**Kinetic properties of glycolaldehyde synthase**

An initial continuous assay included 50 mM potassium phosphate buffer (pH 7.4), 5 mM MgSO_4_, 0.5 mM ThDP, 50 μg mL^-1^ glycerol dehydrogenase, 0.8 mM NADH, and [different](file:///D:\Youdao\Dict\7.5.2.0\resultui\dict\?keyword=different)[concentrations](file:///D:\Youdao\Dict\7.5.2.0\resultui\dict\?keyword=concentrations) [formaldehyde](file:///D:\%E6%9C%89%E9%81%93\Dict\7.2.0.0703\resultui\dict\?keyword=formaldehyde). The reaction was initiated by the addition of purified BFD or mutants at 37 °C, and then an initial linear decrease in absorbance at 340 nm was observed. Enzyme kinetics were determined with [[formaldehyde](file:///D:\%E6%9C%89%E9%81%93\Dict\7.2.0.0703\resultui\dict\?keyword=formaldehyde)](file:///D:\%E6%9C%89%E9%81%93\Dict\7.2.0.0703\resultui\dict\?keyword=formaldehyde) as substrate. The [[formaldehyde](file:///D:\%E6%9C%89%E9%81%93\Dict\7.2.0.0703\resultui\dict\?keyword=formaldehyde)](file:///D:\%E6%9C%89%E9%81%93\Dict\7.2.0.0703\resultui\dict\?keyword=formaldehyde) concentrations ranged from 0.1 to 200 mM. Kinetic parameters *k_cat_* and *K_m_* were estimated by measuring the initial velocities of enzymic reaction and curve-ﬁtting according to the Michaelis-Menten equation, using GraphPad Prism 5 software. All experiments were conducted in triplicate.

**Crystallization and structure refinement of glycolaldehyde synthase**

Crystallization was performed using the hanging drop vapor diffusion method at 16 °C by mixing equal volumes (0.1 μL) of the protein solution (15 mg mL^-1^) and the reservoir solution. Crystals of the GALS protein were grown from drops containing the reservoir solution of 0.2 M calcium acetate, 0.1 M HEPES (pH 7.5) and 40% (w/v) polyethylene glycol (PEG) 400. The crystals were cryoprotected with paratone and flash-frozen in liquid nitrogen. Diffraction data were collected at -175 ℃ at BL19U of Shanghai Synchrotron Radiation Facility (at a wavelength of 0.97853 Å) and processed with HKL-2000 (HKL Research, Inc., Charlottesville, VA, USA)^10^.

The structure of the GALS protein was solved with the molecular replacement (MR) method as implemented in PHENIX^11^ using a structure of benzoylformate decarboxylase (Protein Data Bank [PDB] code 1BFD)^12^ as the search model. Structure refinement was carried out using PHENIX and REFMAC5^13^, and model building was performed using Coot^14^. Stereochemistry of the structure model was analyzed using PROCHECK^15^. Structural analyses were carried out using programs in CCP4^16^ and the PISA server^17^. The statistics of the data collection and structure refinement are summarized (Supplementary Table 3). In brief, the GALS structure at 1.8 Å resolution was refined to Rwork and Rfree values of 0.162 and 0.188, respectively, with 98% of residues in the favored regions of the Ramachandran plot (0 outliers). The PDB ID of our protein is 6A50.

**The computational analysis of glycolaldehyde synthase**

To analyze the structure changes for each mutation of GALS, the backrub module in Rosetta suite^18^ was used to predict the structures of the best mutant with cofactor ThDP of each round. Then the POVME^19^ package was used to calculate the volume changes of the binding pocket. The backrub option file can be found in supplementary parameters.options files. And POVME configuration file can be found in POVME_protocol.ini.

To analyze interaction between the intermediate analogue (IMA) and mutations in GALS, the RosettaLigand application^20^ from Rosetta program suite version 3.5 was used to dock IMA into the crystal structure of GALS. The IMA structure was built based on the geometric orientation and a total of 67 IMA conformers were generated using OpenBabel version 2.4.1^21^. After three docking stages (see Supplementary Dataset: Backrub_protocol), totally 1,000 docking models were generated in the whole simulation process. The docking model with the lowest binding energy (interface_delta_X) was identified as the candidate. All molecular graphics were rendered using UCSF Chimera software version 1.12^4^.

**Phosphoketolase (PK) identification**

To investigate the distribution of PK in bacteria, we predicted all potential PKs in bacteria by searching the NCBI (<https://www.ncbi.nlm.nih.gov/>) bacteria NR database with the conserved protein domain family PRK05261 (https://www.ncbi.nlm.nih.gov/Structure/cdd/cddsrv.cgi?uid=235379). After filtering the redundant sequences by cd-hit with a cutoff 0.9, a total of 2,149 PKs were predicted in bacteria. The PKs are mainly distributed in *Proteobacteria*, *Actinobacteria* and *Firmicutes*, and involved in 111 bacteria families. We then randomly selected a PK in each family, and a Neighbor joining (NJ) tree was constructed based on the 111 PKs. Based on the phylogenetic relationship among 111 families, we divided the 111 PKs into eight classes. Finally, eight PKs were selected from each class to synthesis and functional verification. The accession numbers from PK1 to PK8 are WP 084220650.1, WP 009154258.1, WP 082308260.1, WP 056007231.1, AHG93689.1, OJY14508.1, WP 058186001.1 and WP 011743105.1.

**Expression, purification, and enzyme kinetics of PKs**

The PKs coding genes of different species were ligated into the expression vector pET-28a via *Nde*І and *Xho*I restriction sites to construct the recombinant plasmids. All enzymes were expressed in BL21 (DE3) and purified on a Ni-NTA column as same as BFD. Large-scale purification (800 mL) typically produced about 5-50 mg each enzyme. The protein concentration was determined using a BCA Protein Assay Reagent Kit (Pierce, USA) with BSA as the standard.

The rate of acetyl phosphate production was determined by enzyme coupling. The reaction mixture (200 µL) contained 50 mM potassium phosphate buffer (pH 7.5), 5 mM MgSO4, 1 mM ThDP, 10 mM glycolaldehyde, 1 mM ADP, 0.2 mg mL^-1^ acetate kinase, 5 U hexokinase, 2.5 U Glucose-6-Phosphate Dehydrogenase, 1 mM NADP^+^ and 10 mM glucose. Various PKs (0.5 mg mL^-1^) from different species were added into the reaction system. The reactions were conducted at 37 °C. The production of NADPH was detected at 340 nm. The amount of NADPH produced was equal to the amount of acetyl phosphate produced. Enzyme kinetics were determined with glycolaldehyde as substrate. The glycolaldehyde concentrations ranged from 0.1 to 110 mM. Kinetic parameters were determined using GraphPad Prism 5 (GraphPad Software, USA).

**Computational modeling for ACPS**

The computational model was constructed with SWISS-MODEL (<http://swissmodel.expasy.org>)^22-25^, based on the crystal structure (PDB code: 3AHE), and the binary complex of ACPS with ThDP and substrate was generated with PyMOL^26^. The model contains 169 atoms with a total charge of 0, including the side chains of His64, His553, Glu479, Tyr501, His142, Gly155, Glu437, Asn549 and His97, the substrate and the cofactor ThDP. According to the previous study^27^, since His553 is considered as the most possible candidate of proton donor, the His553 was modeled in the doubly protonated state. Generally, the glutamate (Glu479) is modeled in protonated state for forming a hydrogen bond with the N1’ atom of ThDP. According to the interaction mode of pocket residues, His64, His142, His97 were modeled in their singly protonated states, and Glu437 was set to its deprotonated state. All calculations were performed with Gaussian 03^28^ program package using hybrid density functional theory method. Geometrical structures were optimized at B3LYP/6-31G (d, p) level.

In the reactant model, the substrate is stabilized by hydrogen bonds with His553 and ThDP. Furthermore, the substrate locates in suitable position for nucleophilic attack where the distance between the C of ThDP and carbonyl carbon of substrate is 3.95 Å. The transition state 1 (TS1) corresponds to the transition state of the first step, in which the distance between C of ThDP and carbonyl C changes from 3.95 Å in Reactant to 2.70 Å in TS1. Meanwhile, the distance between O of substrate and H of His553 has shortened to 1.60 Å in TS1. From the energy profile, the obtained intermediate 1 (IM1) is stable relative to Reactant. For the dehydrogenation reaction assisted by ThDP, the N4’ should be firstly deprotonated by Glu437. The generated intermediate 2 (IM2) is calculated to be more stable than IM1 by 5.67 kcal mol^-1^. Starting from IM2, the optimized transition state 2 (TS2) is an eight-membered ring, in which the distance between C and H is 1.33 Å and the distance between H and N4’ is 1.40 Å. The calculated energy barriers for the formation of IM1 and product (DHEThDP) are 11.36 and 15.13 kcal mol^-1^, respectively. The catalytic process was [described](javascript:void(0);) in Supplementary Fig. 13.

**The synthesis of acetyl-CoA from formaldehyde *in vitro***

The synthesis of glycolaldehyde from [formaldehyde](file:///D:\%E6%9C%89%E9%81%93\Dict\7.2.0.0703\resultui\dict\?keyword=formaldehyde) was executed by adding different concentrations [formaldehyde](file:///D:\%E6%9C%89%E9%81%93\Dict\7.2.0.0703\resultui\dict\?keyword=formaldehyde). The 1 mL reaction system contained 50 mM potassium phosphate buffer (pH 7.5), 5 mM MgSO_4_, 1 mM ThDP and 2 mg mL^-1^ GALS. The reaction was conducted at 37 °C for 2 hours. Then, 200 μL of sample was taken out and added 200 μL acetonitrile to quench the reaction. The yield of glycolaldehyde was d[etected](javascript:void(0);) by HPLC.

The synthesis of acetic [acid](javascript:void(0);) from glycolaldehyde was carried out by adding different concentrations glycolaldehyde. In addition, the influence of [formaldehyde](file:///D:\%E6%9C%89%E9%81%93\Dict\7.2.0.0703\resultui\dict\?keyword=formaldehyde) to ACPS activity was realized by adding extra [formaldehyde](file:///D:\%E6%9C%89%E9%81%93\Dict\7.2.0.0703\resultui\dict\?keyword=formaldehyde). The 2 mL reaction system contained 50 mM potassium phosphate buffer (pH 7.5), 5 mM MgSO_4_, 1 mM ThDP, 1 mM ADP, 0.2 mg mL^-1^ acetate kinase, 10 U hexokinase, 20 mM glucose, 2 mg mL^-1^ ACPS. Then, 200 μL of sample was taken out and added 200 μL acetonitrile to quench the reaction at each time point. The yield of [acetic](javascript:void(0);) [acid](javascript:void(0);) was d[etected](javascript:void(0);) by HPLC. HPLC conditions: column: Aminex HPX-87H (Bio-Rad); detection wavelength: 210 nm; mobile phase: 5 mM sulphuric acid; flow rate: 0.6 mL min^-1^; sample volume: 20 μL; column temperature: 40 °C.

One-carbon assimilation pathway *in vitro* from formaldehyde to acetyl-CoA was constructed through subsequent methods. A 2 mL reaction system contained 50 mM triethanolamine buffer (pH 7.5), 5 mM MgSO_4_, 1 mM ThDP, 1 g L^-1^ [formaldehyde](file:///D:\%E6%9C%89%E9%81%93\Dict\7.2.0.0703\resultui\dict\?keyword=formaldehyde), 20 mM CoA, 10 mM K_3_PO_4_, 2 mg mL^-1^ GALS, 2 mg mL^-1^ ACPS, 0.5 mg mL^-1^ PTA. The reaction was conducted at 37 °C. 200 μL of sample was taken out and added 200 μL acetonitrile to quench the reaction at each time point. Sample was d[etected](javascript:void(0);) by HPLC. HPLC analysis for acetyl-CoA samples was detected at 254 nm by the ZORBAX-SB-Aq (Agilent) column at a flow rate of 0.3 mL min^-1^. The buffers were 0.2 M potassium phosphate buffer (pH 4.8) (solution A) and pure acetonitrile (solution B). The HPLC method was 0-10 min with 3% solution B, 10-25 min with 3-20% solution B, 25-30 min with 20% solution B, 30-31 min with 20%-3% solution B, 31-39 min with 3% solution B.

**Liquid Chromatography-Mass Spectrometry (LC-MS) analysis for ^13^C-labeled metabolites**

Recombinant plasmid, pET-28a-*GALS*-*ACPS*-*PTA*, was constructed using different enzyme cutting sites and transformed into *E. coli* BL21 (DE3). The expression of the gene of interest was executed as well as BFD. The clarified cellular lysates were prepared by using high-pressure homogenizer to lyse cells (JNBIO, China) and centrifuging at 10,000 g for 60 min at 4 °C. The reaction was started by adding 1 mM [Coenzyme](file:///D:\Youdao\Dict\7.5.2.0\resultui\dict\?keyword=coenzyme)[A](file:///D:\Youdao\Dict\7.5.2.0\resultui\dict\?keyword=A) (CoA) and 0.3 g L^-1^ ^13^C-labeled [formaldehyde](file:///D:\%E6%9C%89%E9%81%93\Dict\7.2.0.0703\resultui\dict\?keyword=formaldehyde) in clarified cellular lysates. The reaction was conducted at 37 °C for 2 hours and then two times the volume of acetonitrile was added to quench the reaction. Sample was enriched by the cryoconcentration to 100 μL and d[etected](javascript:void(0);) by LC-MS. The procedures of various control experiments were consistent with the above.

The strain containing bi-plasmid (*BsMDH*-pCDF, *GALS-ACPS-PTA*-pET-28a) or the strain containing bi-plasmid (*BsMDH*-pCDF, pET-28a) was used for ^13^C-labeled metabolic tracer analysis *in vivo.* The cells that had induced the protein in LB were re-inoculated into M9 medium containing 8 g L^-1^ ^13^C-labeled methanol. Cells from different culture times were collected and used to detect intracellular metabolites and proteinogenic amino acids. For intracellular metabolites, 10 mL of cells were collected after 10 hours, and then intracellular metabolites were extracted with a quenching reagent (methanol: acetonitrile: H_2_O = 6:2:2, -20 °C). For proteinogenic amino acids, 3 mL of cells were harvested and resuspended in 6 M HCl at 95 °C for 24 h to hydrolyze the biomass proteins into amino acids. Samples were concentrated and tested with LC-MS.

^13^C-labeled metabolites were detected by LC-MS. A UHPLC LC-30A system (Shimadzu, Japan) equipped with a Triple TOF 5600 mass spectrometer (Sciex, USA) was used for LC-MS/MS analysis. The samples were separated by a zic-HILIC column (100 mm × 2.1 mm, 3.5 μm) (Merck, German). Solvents were composed of water/acetonitrile/ ammonium acetate (A: 100%/0%/10 mM, B: 0%/100%/0 mM). The LC method was 0-3 min with 90% B, 3-25 min with 90-60% B, 25-30 min with 60% B, 30-38 min with 90% B. A flow rate of 0.3 mL min^-1^ was employed. The MS parameters were as follows: ESI source; negative mode; ion voltage 4500 V; declustering potential 80 V; source temperature 600 °C; curtain gas, 35 psi; nebulizer gas 55 psi; heater gas 55 psi. Each scan cycle contained one TOF MS survey scan and 15 MS/MS scans. The mass ranges were m/z 50-1200 for TOF MS and m/z 30-1200 for MS/MS. Acquisition of MS/MS spectra was controlled by IDA function of the Analyst TF 1.6 software (Sciex, USA) with dynamic background subtraction. Mass accuracy was calibrated by automated calibrant delivery system (Sciex, USA) interfaced to the second inlet of the DuoSpray source. Calibration was performed for every five samples.

**Supplementary Figures**


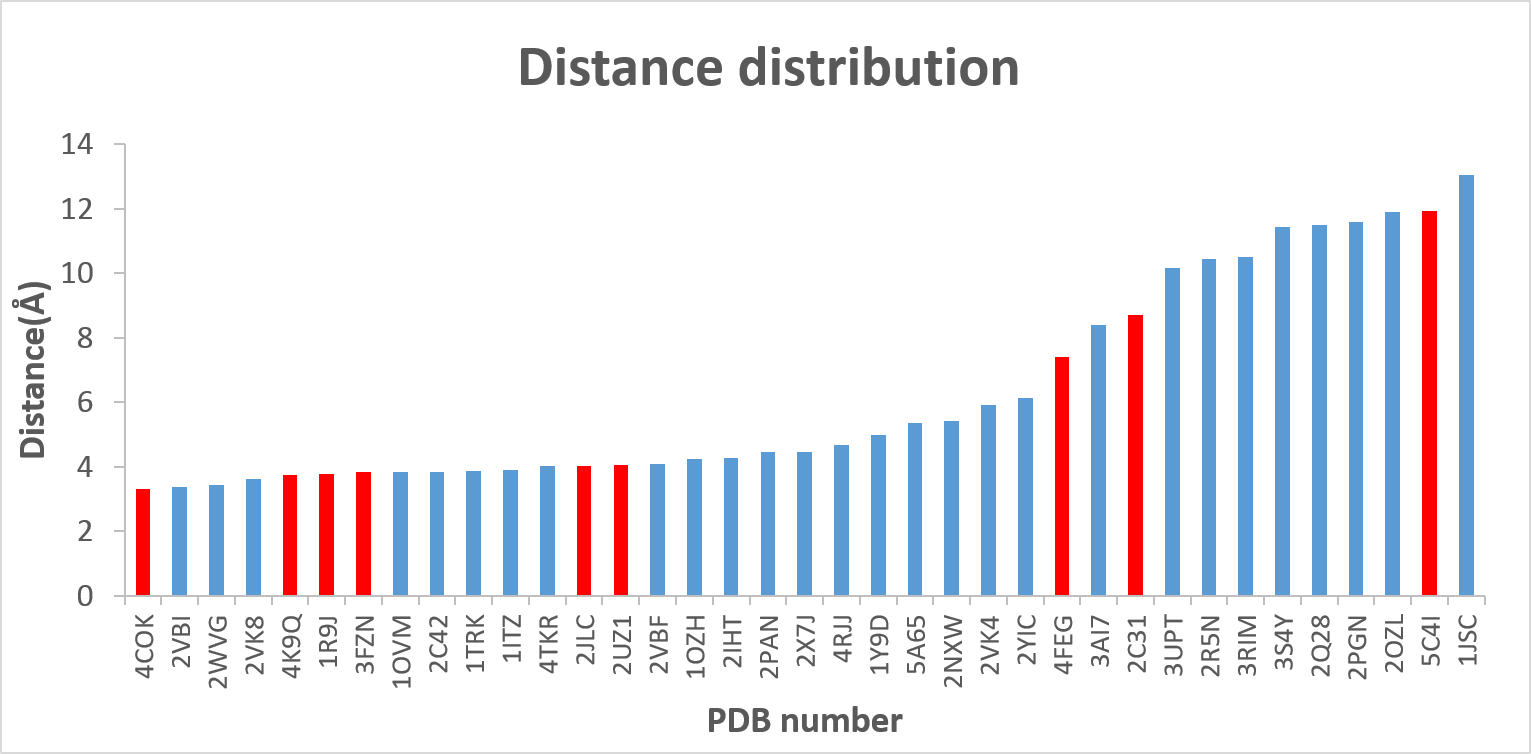


**Supplementary Figure 1. The distribution of average distances of the screened protein structures.** The average distances were calculated between C2 atom in ThDP and C1 & C2 in glycolaldehyde. Six candidates with short distance and clear functional annotation were selected for the next experimental validation. Three proteins with long distances were randomly chosen as controls. All the selected proteins were colored in red.


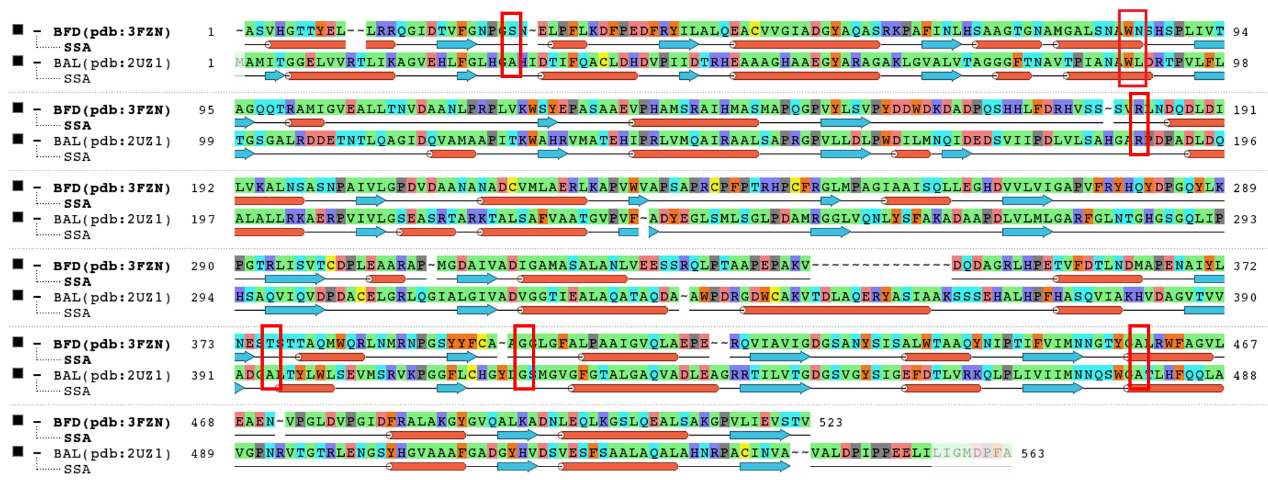


**Supplementary Figure 2. The sequence alignment between BFD and BAL**. The mutations (A28I, W89R-L90T, R188H, A394G, G419N and A480W) in BAL have been proved to be able to improve activities of formose reaction^29^. The corresponding residues in BFD are selected (the red boxes).


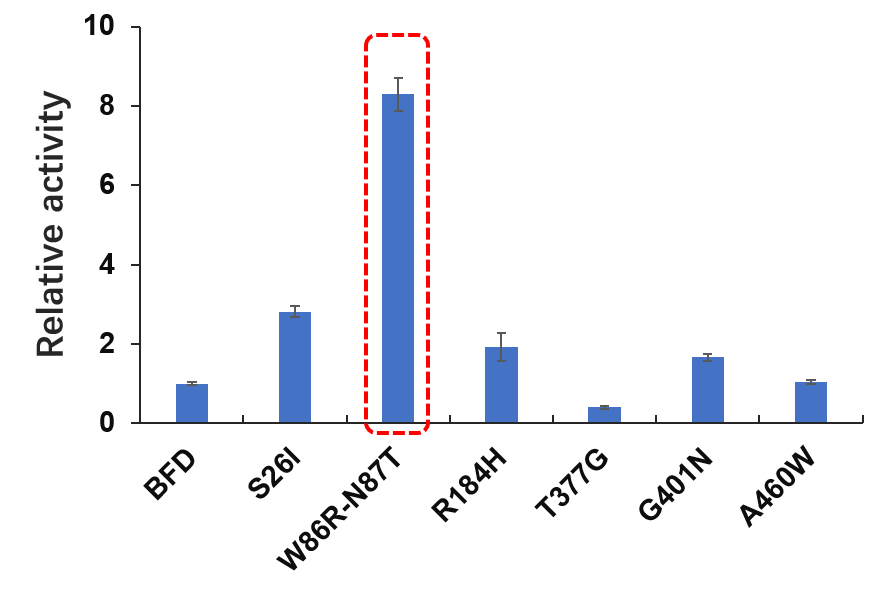


**Supplementary Figure 3. Experimental validation of mutations in BFD**. The protein expression of BFD and its mutants were executed as well as we described above. 10 OD cells in 1 mL lysis buffer were used to test the activities by adding 2 g L^-1^ formaldehyde. The yield of glycolaldehyde was determined by HPLC. The relative activity is equal to the ratio of the amount of glycolaldehyde in the mutants to that in BFD. Error bars represent s.d. (standard deviation), *n*=3. Source data are provided as a Source Data file.


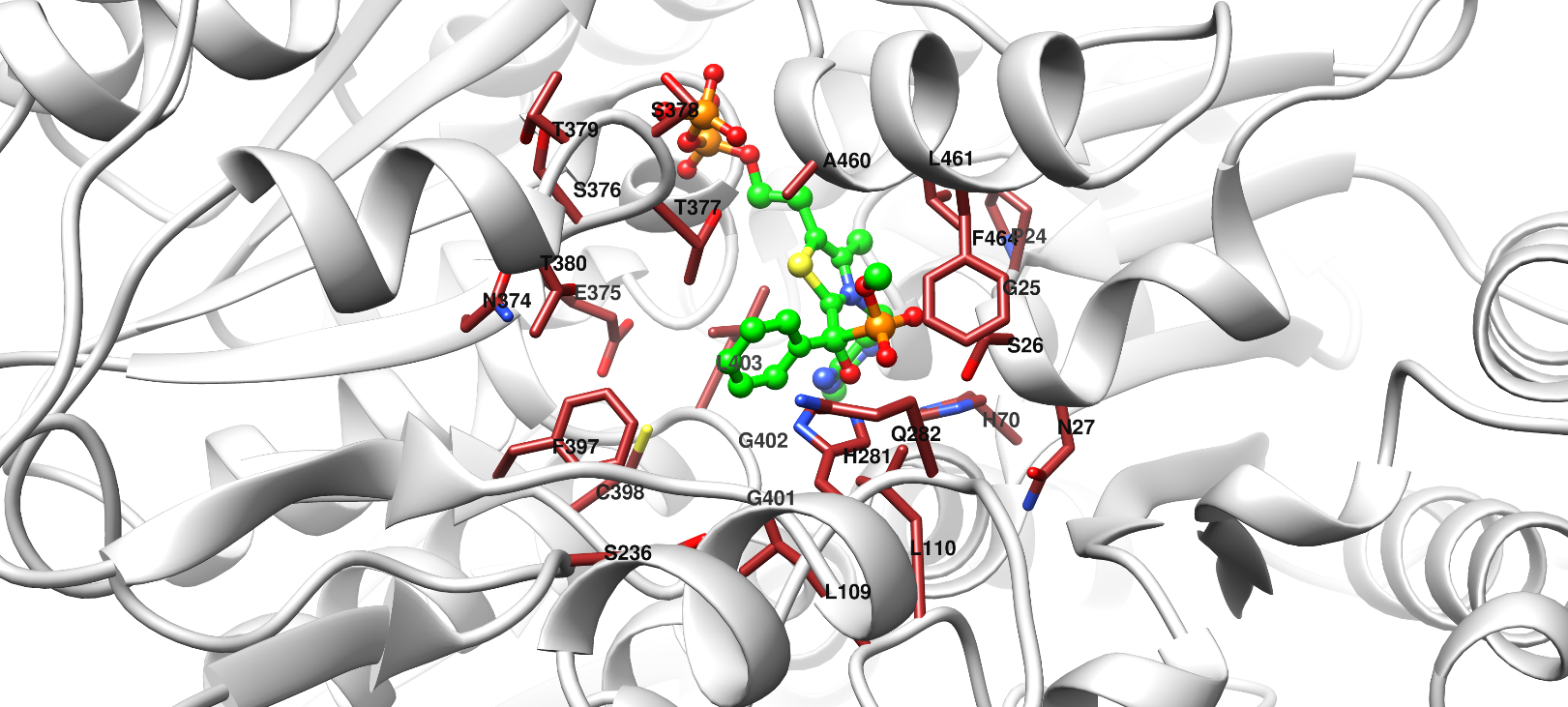


**Supplementary Figure 4.** **Selection of residues for single-point saturation mutation**. The residues within 8Å distance from benzene ring of the intermediate analogue were shown in brown and ThDP was shown in green. All residues in brown were selected to do single-point saturation mutation.


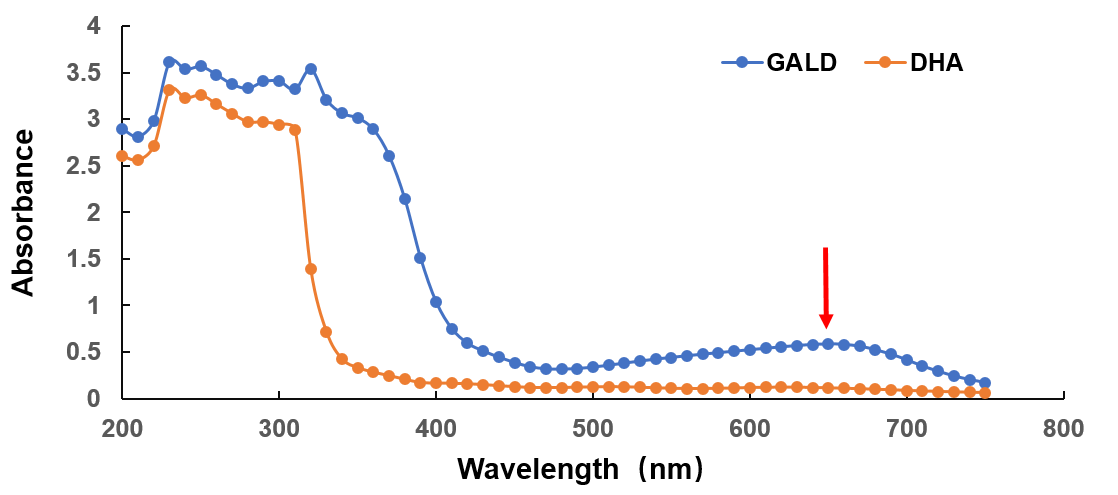


**Supplementary Figure 5.**  **Full-wavelength scanning for glycolaldehyde and 1, 3-dihydroxyacetone with diphenylamine solution**. The full-wavelength scanning was performed after 0.5 g L^-1^ GALD or DHA reacting with diphenylamine solution. The red arrow indicates the selected wavelength for subsequent screening tests. GALD: glycolaldehyde; DHA: 1, 3-dihydroxyacetone. Source data are provided as a Source Data file.

**
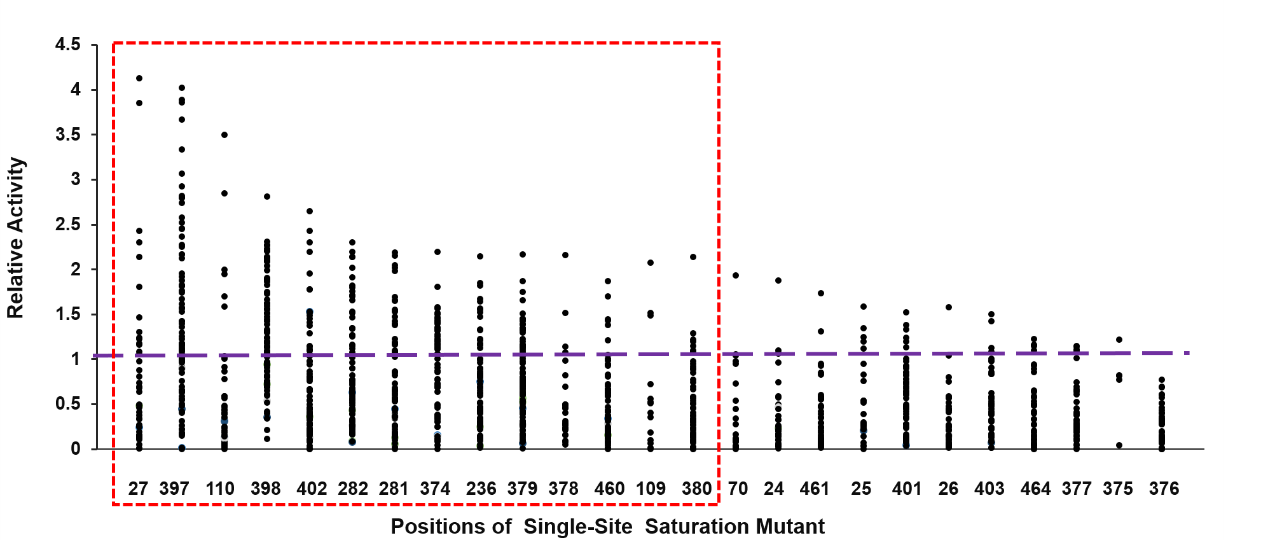
**

**Supplementary Figure 6.**  **Experimental validation of single-point saturation mutant**. The x-axis label represents the selected positions in BFD. The single-point saturation mutation assays were carried out for each selected position. To cover all kinds of amino acids mutation, we selected 90 different mutants at each position.The y-axis label represents the relative catalytic activity of different mutants. The relative activity was defined as the ratio of the production of glycolaldehyde in the mutants to that in M1 (BFD contains mutations W86R and N87T). The yields of glycolaldehyde were determined by the chromogenic reaction. 14 positions containing higher activity mutants were for selected for the next round of experiments in the red rectangle. Source data are provided as a Source Data file.


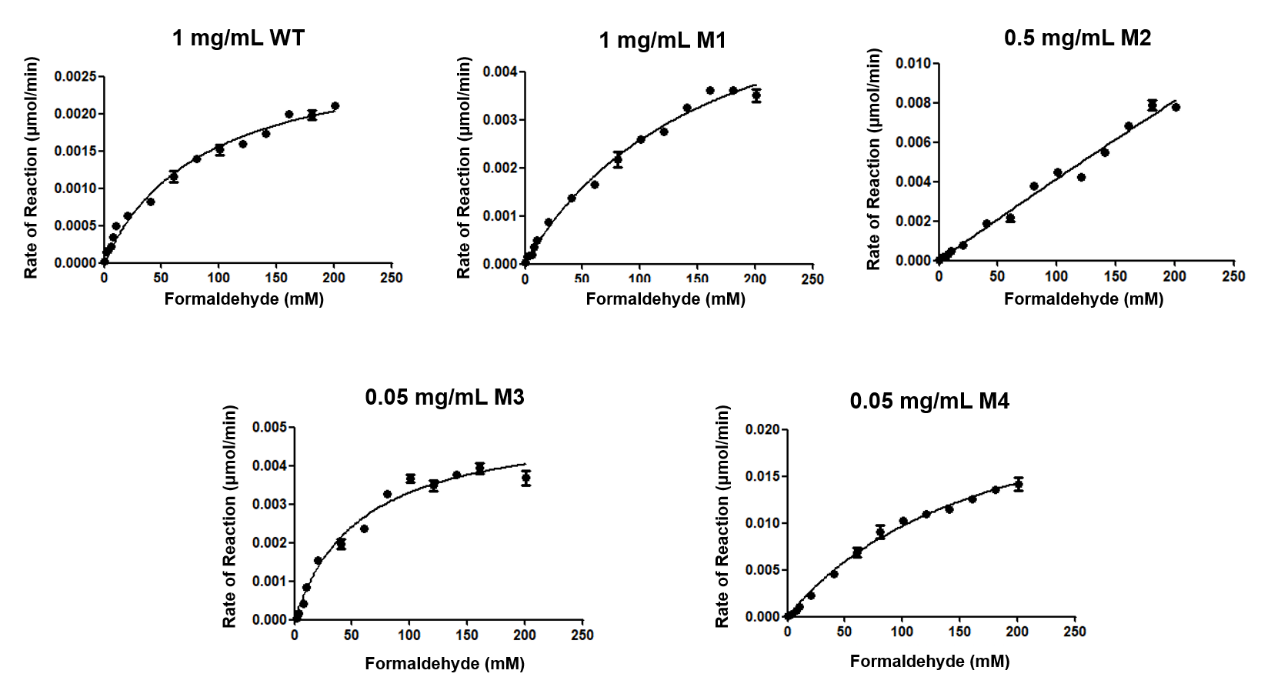


**Supplementary Figure 7.** **Enzyme kinetics of BFD-WT and mutants**. Enzyme kinetics were determined with 1 mg mL^-1^ (WT or M1)、0.5 mg mL^-1^ (M2)、0.05 mg mL^-1^ (M3 or M4) enzyme. The concentration of formaldehyde ranged from 0.1 to 200 mM. Error bars represent s.d. (standard deviation), *n*=3. Source data are provided as a Source Data file.

**
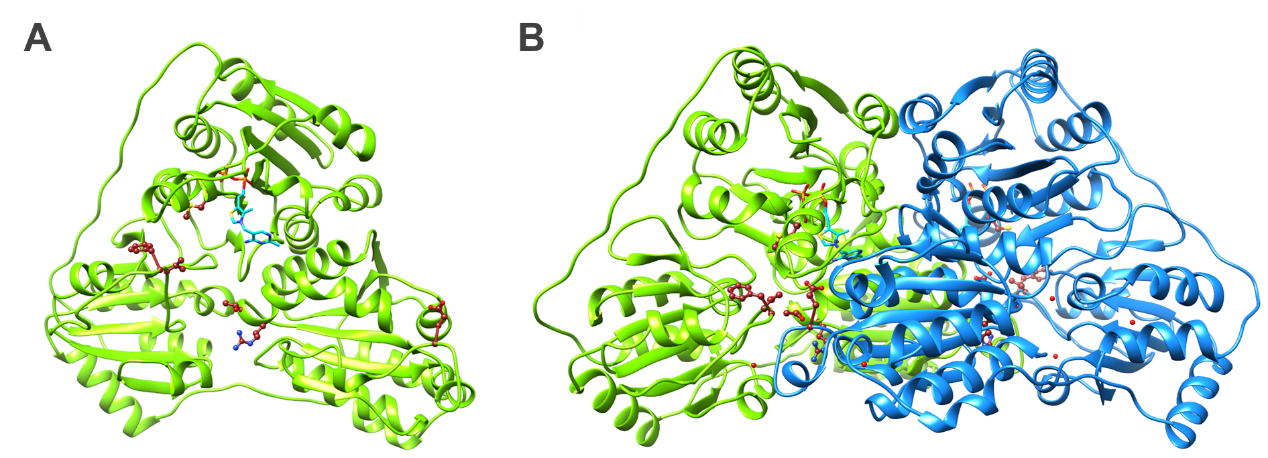
**

**Supplementary Figure 8.**  **Crystal structure of GALS**. **(A)** Overview image of the crystal structure of GALS chain A. This image depicts the relative positions of seven mutations (brown) that were engineered in native BFD. **(B)** Overview image of the crystal structure of GALS chain A and B. The GALS catalyzes as a homodimer. The active pocket located at the dimer interface^30^.


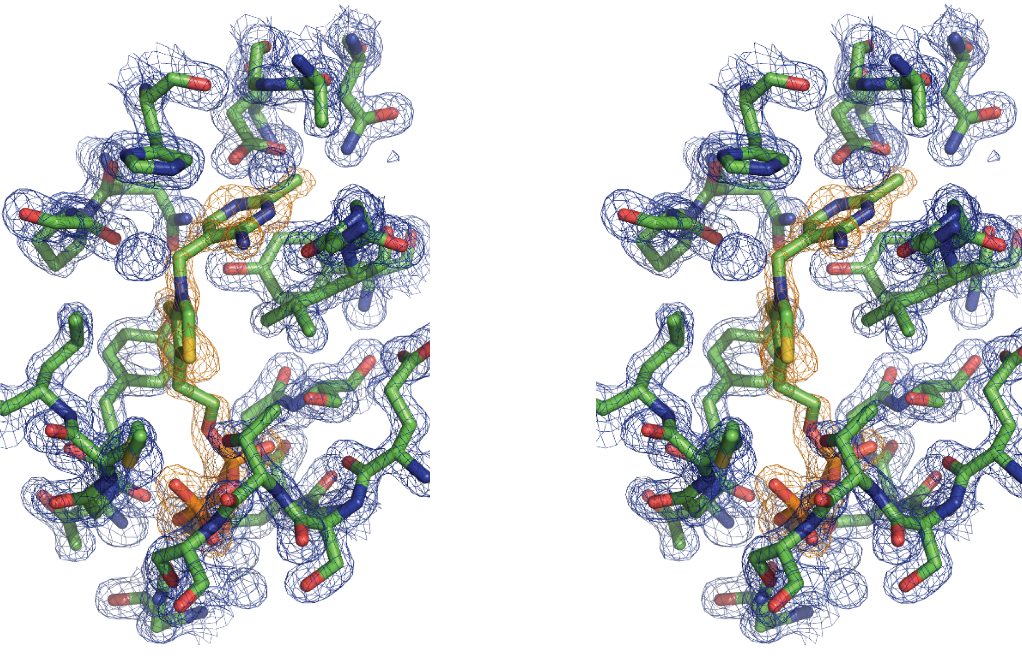


**Supplementary Figure 9.** **Stereo view showing the ThDP and surrounding residues.** The 2Fo-Fc electron density map of the ThDP is colored in orange, and the 2Fo-Fc electron density map of surrounding residues is colored in blue. Both maps are contoured at 1.5 sigma level.

**Supplementary Figure 10.** **The phylogenetic tree of PKs**. The phylogenetic relationship of phosphoketolases among 111 bacteria families. The NJ tree was constructed based on 111 phosphoketolases which were selected from 111 bacteria families. All nodes received bootstrap support values from 100 replicates. The selected eight PKs for the next experiments were highlighted using different colours.


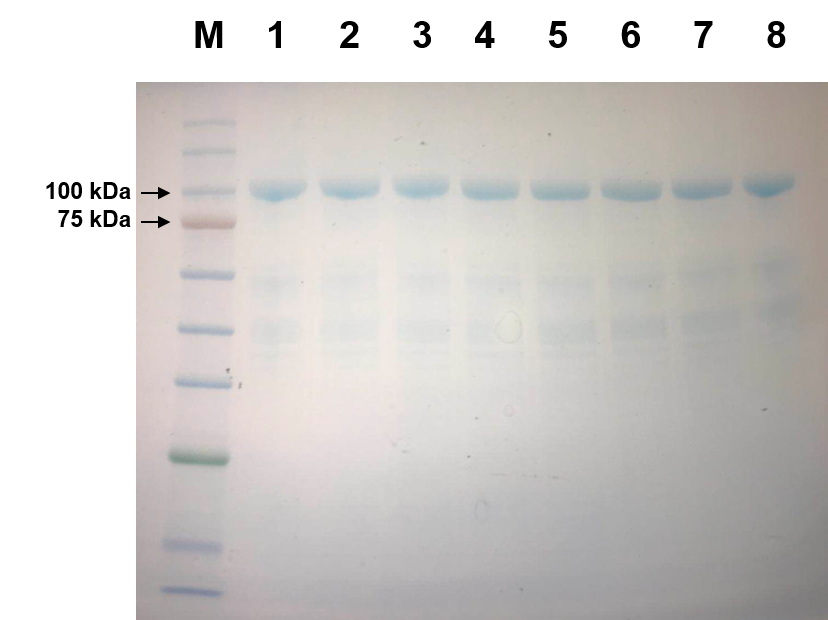


**Supplementary Figure 11.**  **SDS-PAGE of the purified PKs.**M: protein marker; 1: PK1; 2: PK2; 3: PK3; 4: PK4; 5: PK5; 6: PK6; 7: PK7; 8: PK8. The experiment was executed using 0.5 mg mL^-1^ enzymes.


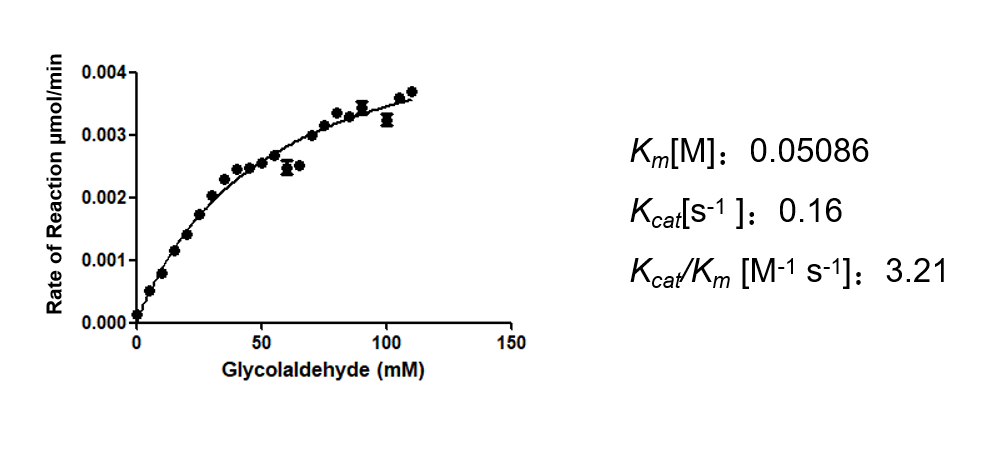


**Supplementary Figure 12.** **Enzyme kinetics of ACPS**. Enzyme kinetics were determined using 0.25 mg mL^-1^ ACPS and different concentrations glycolaldehyde. The glycolaldehyde concentrations ranged from 0.1 to 110 mM. Error bars represent s.d. (standard deviation), *n*=3. Source data are provided as a Source Data file.


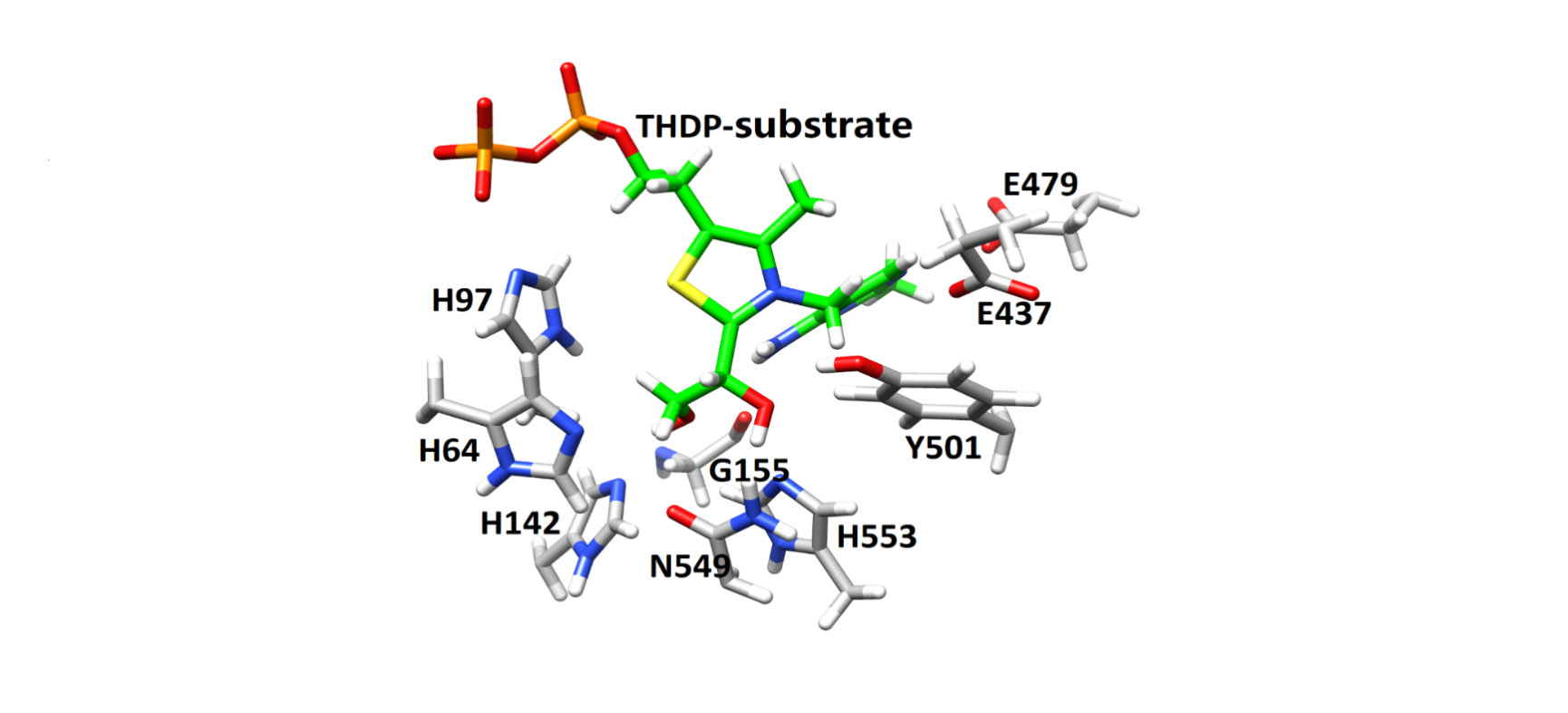


**Supplementary Figure 13.** **Computational model for calculation**. The model contains 169 atoms with a total charge of 0, including the side chains of His64, His553, Glu479, Tyr501, His142, Gly155, Glu437, Asn549 and His97, the substrate and the cofactor ThDP.

**Supplementary Figure 14.** **Proposed mechanism of ACPS**. His553 was considered as the most possible proton donor for the formation of IM1. The formation of IM2 from IM1 needs the assistance of Glu479 and Glu437. N4’ of ThDP acts as proton acceptor for the formation of DHEThDP. H97 acts as proton donor for the dehydration process of DHEThDP. His64, Tyr501 and Asn549 are important for the nucleophilic attack by “Pi” and they together form the binding site of “Pi”.


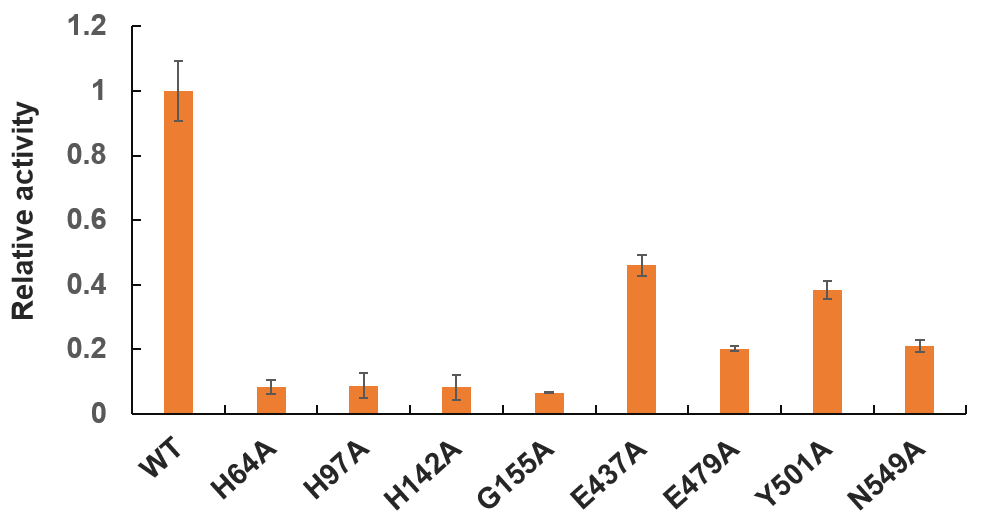


**Supplementary Figure 15.** **Validation of the important residues in ACPS**. The relative activity is equal to the ratio of the production of acetyl phosphate in the mutants to that in WT. The rate of acetyl phosphate production was determined by enzyme coupling. The standard reaction mixture (200 µL) contained 50 mM potassium phosphate buffer (pH 7.5), 5 mM MgSO_4_, 1 mM ThDP, 10 mM glycolaldehyde, 1 mM ADP, 0.2 mg mL^-1^ acetate kinase, 5 U hexokinase, 2.5 U Glucose-6-Phosphate Dehydrogenase, 1 mM NADP^+^ and 10 mM glucose. WT or different mutants (0.5 mg mL^-1^) were added into the reaction system. The reactions were conducted at 37 °C. The production of NADPH was detected at 340 nm. The amount of NADPH produced was equal to the amount of acetyl phosphate produced. Error bars represent s.d. (standard deviation), *n*=3. Source data are provided as a Source Data file.


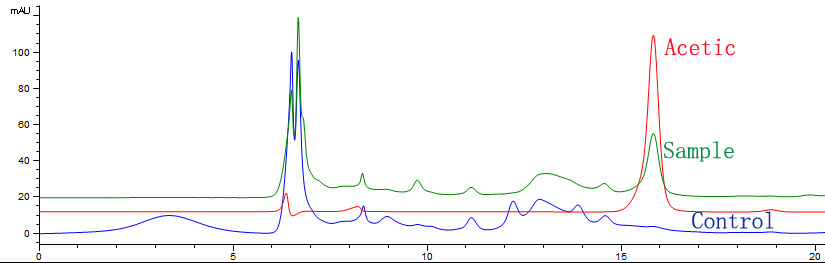


**Supplementary Figure 16.** **HPLC chromatogram of acetic acid**. The red line represents acetic acid standard. The green line represents reaction sample with 0.5 mg mL^-1^ ACPS. The blue line represents control without ACPS. The experiment was executed using 10 mM glycolaldehyde at 37 °C for 2 hours. The 1 mL reaction system also contained 50 mM potassium phosphate buffer (pH 7.5), 5 mM MgSO_4_, 1 mM ThDP, 1 mM ADP, 0.2 mg mL^-1^ acetate kinase, 10 U hexokinase, and 20 mM glucose.


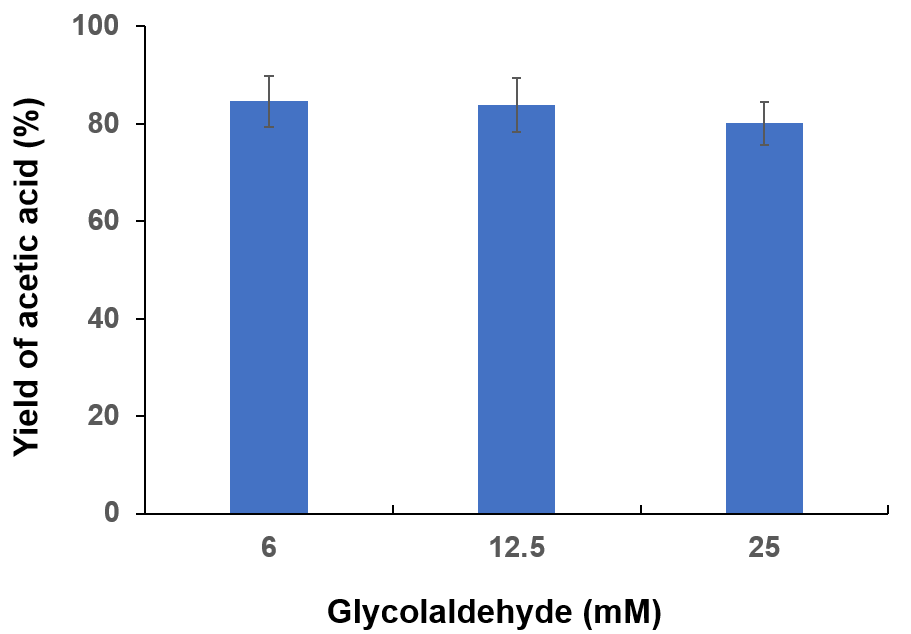


**Supplementary Figure 17.** **The yield of acetic acid from glycolaldehyde**. The experiment was carried out by adding 2 mg mL^-1^ purified ACPS. The reaction was conducted at 37 °C for 2 hours. Samples were analyzed by HPLC. Error bars represent s.d. (standard deviation), *n*=3. Source data are provided as a Source Data file.


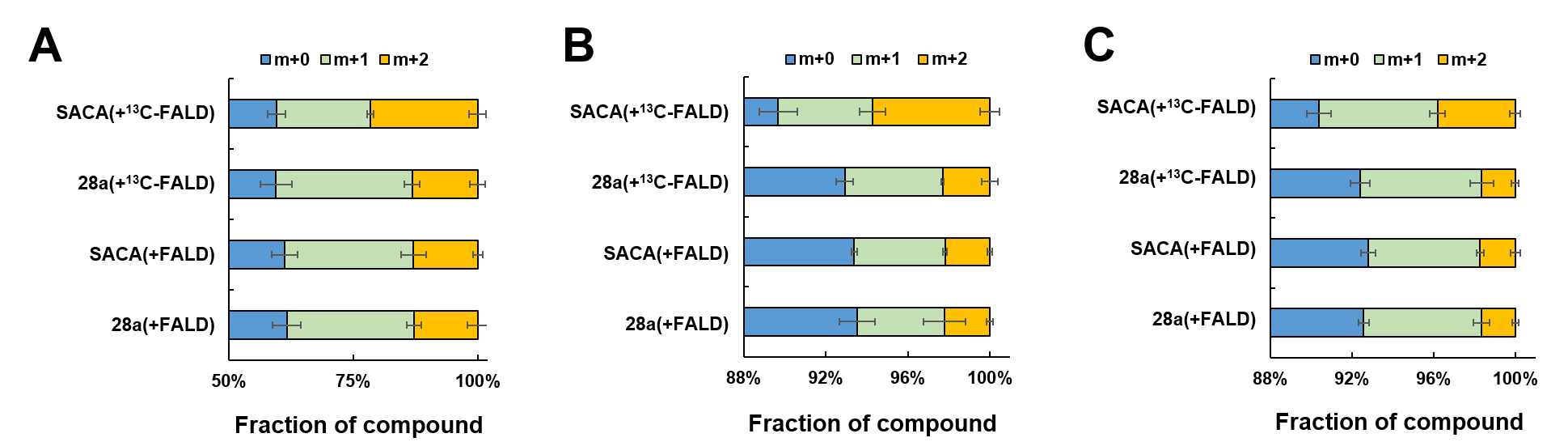


**Supplementary Fig. 18 ^13^C-labeled metabolic tracer analysis of the SACA pathway in cellular lysates. (A), (B) and (C)** indicate the relative abundance of acetyl-CoA, fumarate and [malate](javascript:void(0);), respectively. All assays were performed in cellular lysates by adding 1 mM CoA. SACA: the strain contains the vector *GALS-ACPS-PT*A-28a; 28a: the strain contains the empty vector of 28a; FALD: formaldehyde; ^13^C-FALD: ^13^C labeled formaldehyde; m+0: without ^13^C labelling; m+1: single ^13^C labelling; m+2: double ^13^C labelling. Error bars represent s.d. (standard deviation), *n*=3. Source data are provided as a Source Data file.

**
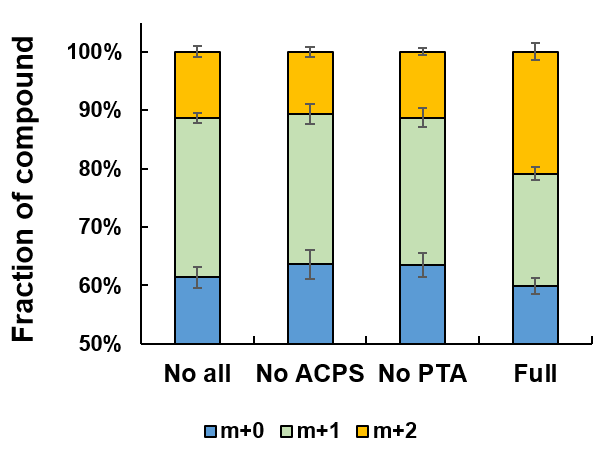
**

**Supplementary Figure 19.** **Relative abundance of acetyl-CoA**. The experiment was carried out in different strains in LB medium by feeding ^13^C-labeled [formaldehyde](file:///D:\%E6%9C%89%E9%81%93\Dict\7.2.0.0703\resultui\dict\?keyword=formaldehyde). No all: the strain contains the empty vector 28a; No ACPS: the strain contains the SACA pathway without ACPS; No PTA: the strain contains the SACA pathway without PTA; Full: the strain contains the SACA pathway. m+0: without ^13^C labelling; m+1: single ^13^C labelling; m+2: double ^13^C labelling. Error bars represent s.d. (standard deviation), *n*=3. Source data are provided as a Source Data file.


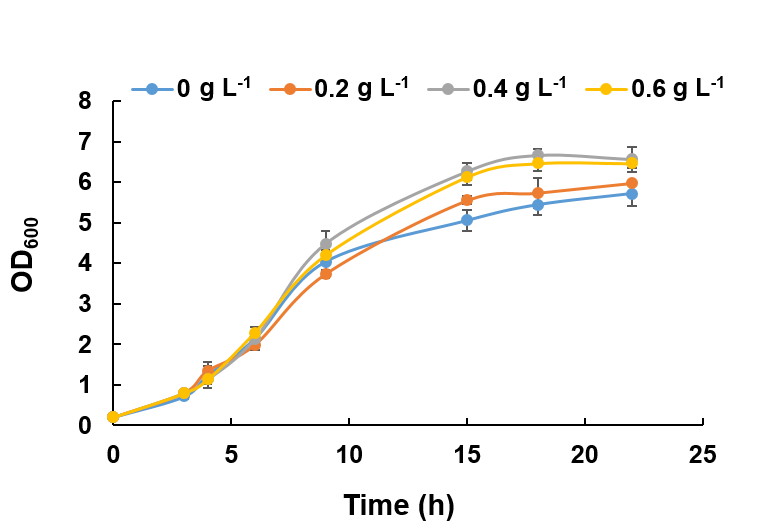


**Supplementary Figure 20.** **Cell growth using glycolaldehyde as supplemental carbon source.** Cells were incubated in LB medium. IPTG was added at 3 hour for protein expression induction. The different concentrations of glycolaldehyde were added at 4 hour. Error bars represent s.d. (standard deviation), *n*=3. Source data are provided as a Source Data file.


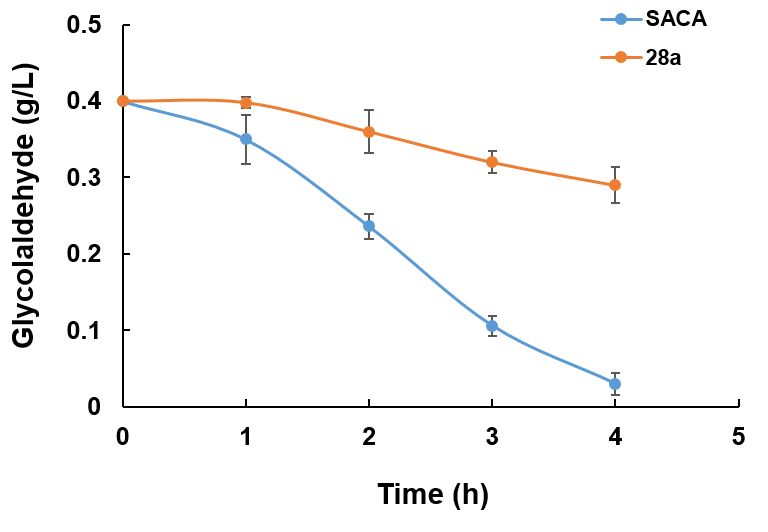


**Supplementary Figure 21. Glycolaldehyde consumption.** Cells were incubated in LB medium and IPTG was added at 3 hour to induce protein expression. 0.4 g L^-1^ glycolaldehyde was fed at 4 hour. Then the rest of glycolaldehyde was detected by the HPLC. Different points represent the glycolaldehyde concentration at different times after glycolaldehyde addition. SACA: the strain contains the vector GALS-ACPS-PTA-28a; 28a: the strain contains the empty vector of 28a. Error bars represent s.d. (standard deviation), *n*=3. Source data are provided as a Source Data file.


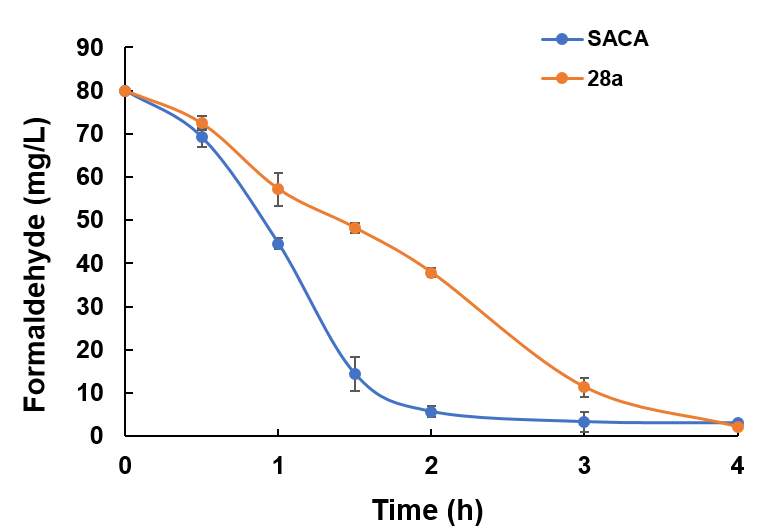


**Supplementary Figure 22.** **Formaldehyde consumption.** Cells were incubated in LB medium and IPTG was added at 3 hour to induce protein expression. 80 mg L^-1^ [formaldehyde](file:///D:\%E6%9C%89%E9%81%93\Dict\7.2.0.0703\resultui\dict\?keyword=formaldehyde) was fed at 4 hour. And then the rest of formaldehyde was detected by the colorimetric NASH reaction every half of hour. Different points represent the formaldehyde concentration at different times after formaldehyde addition. SACA: the strain contains the vector *GALS-ACPS-PT*A-28a; 28a: the strain contains the empty vector of 28a. Error bars represent s.d. (standard deviation), *n*=3. Source data are provided as a Source Data file.


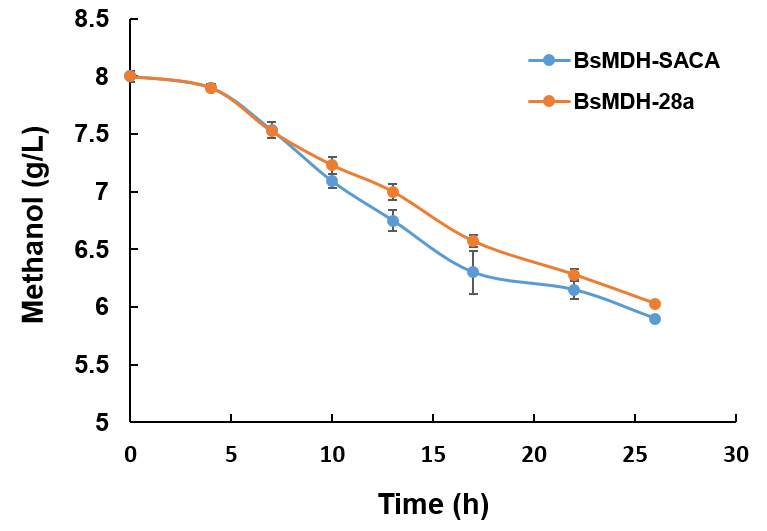


**Supplementary Figure 23. Methanol consumption.** Cells initially were cultured in LB medium at 37 °C; 0.5 mM IPTG was added when OD_600_ is ~ 0.6; after 1 hour incubation, cells were transferred to M9 minimal medium (1% v/v) by adding 1 g L^-1^ yeast extract, 2 g L^-1^ tryptone, 0.1 mM IPTG, trace elements and 8 g L^-1^ methanol. Methanol was quantified via HPLC as described by analysing of the culture supernatant^31^. BsMDH-SACA: the strain contains both the vector BsMDH-pCDF and GALS-ACPS-PTA-28a; BsMDH-28a: the strain contains both BsMDH-pCDF and the empty vector 28a. Error bars represent s.d. (standard deviation), *n*=3. Source data are provided as a Source Data file.


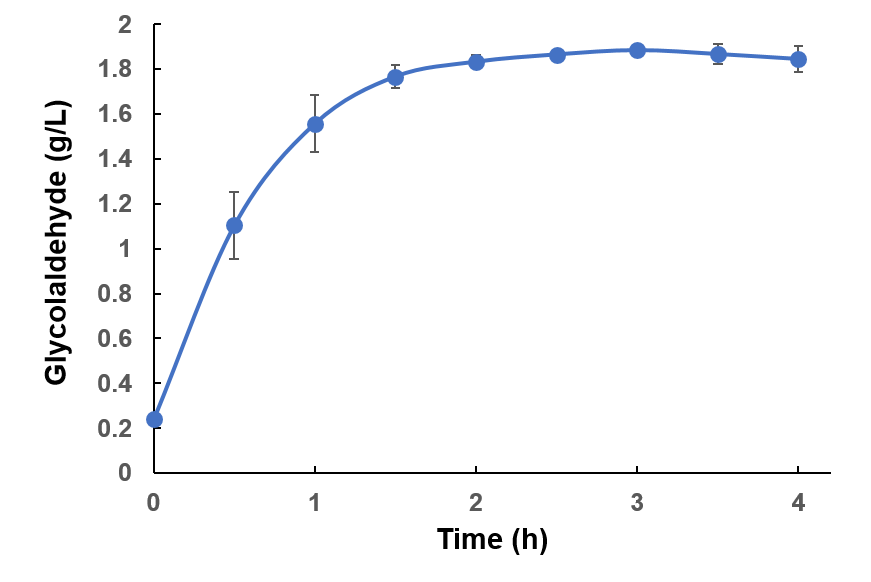


**Supplementary Figure 24. The biosynthesis of glycolaldehyde from formaldehyde.** The experiment was carried out by adding 1 mg mL^-1^ of the purified GALS at 37 °C with 2 g L^-1^ of formaldehyde. Different points represent the glycolaldehyde concentration at different times. Error bars represent s.d. (standard deviation), *n*=3. Source data are provided as a Source Data file.

**Supplementary Table 1. The thermodynamic data of three new designed pathways.**

| **SACA pathway** | ΔrG'^m^ (kJ mol^-1^) | MDF(kJ mol^-1^) | |
| --- | --- | --- | --- |
| 2 Formaldehyde <=> Glycolaldehyde | -27.9 |  | |
| Glycolaldehyde + Phosphate <=> Acetyl phosphate + H_2_O | -59 |  | |
| Acetyl phosphate + CoA <=> Acetyl-CoA + Phosphate | -9.8 |  | |
| **Overall reaction**: [CoA + 2 Formaldehyde <=> Acetyl-CoA + H_2_O](http://equilibrator.weizmann.ac.il/reaction?reactantsId=C00010&reactantsCoeff=-1&reactantsName=CoA&reactantsPhase=aqueous&reactantsConcentration=0.001&reactantsId=C00024&reactantsCoeff=1&reactantsName=Acetyl-CoA&reactantsPhase=aqueous&reactantsConcentration=0.001&reactantsId=C00067&reactantsCoeff=-2&reactantsName=Formaldehyde&reactantsPhase=aqueous&reactantsConcentration=0.001&reactantsId=C00001&reactantsCoeff=1&reactantsName=H2O&reactantsPhase=liquid&reactantsConcentration=1&ph=7.000000&pmg=14.000000&ionic_strength=0.100000&e_reduction_potential=0.000000&max_priority=0&mode=BA) | -96.7 | 26.9 | |
| **FLS pathway** | ΔrG'^m^ (kJ mol^-1^) | | MDF(kJ mol^-1^) |
| 3 Formaldehyde <=> 1,3-Dihydroxyacetone | -45.6 |  | |
| ATP + Glycerone <=> ADP + Glycerone phosphate | -13.7 |  | |
| Glycerone phosphate <=> D-Glyceraldehyde-3-phosphate | 5.5 |  | |
| Pi + NAD^+^ + D-Glyceraldehyde-3-phosphate <=> NADH + 1,3-Bisphosphoglycerate + H^+^ | 24.9 |  | |
| ADP + 1,3-Bisphosphoglycerate <=> ATP + D-Glycerate-3-phosphate | -18.5 |  | |
| D-Glycerate-3-phosphate <=> D-Glycerate-2-phosphate | 4.2 |  | |
| D-Glycerate-2-phosphate <=> Phosphoenolpyruvate + H_2_O | -4.1 |  | |
| ADP + Phosphoenolpyruvate <=> Pyruvate + ATP | -27.7 |  | |
| Pyruvate + CoA + NAD^+^ <=> Acetyl-CoA + CO_2_ + NADH + H^+^ | -35.3 |  | |
| **Overall reaction**: [2 NAD^+^ + ADP + Orthophosphate + CoA + 3 Formaldehyde <=> ATP + 2 NADH + CO_2_ + Acetyl-CoA + H_2_O](http://equilibrator.weizmann.ac.il/reaction?reactantsId=C00002&reactantsCoeff=1&reactantsName=ATP&reactantsPhase=aqueous&reactantsConcentration=0.001&reactantsId=C00003&reactantsCoeff=-2&reactantsName=NAD+&reactantsPhase=aqueous&reactantsConcentration=0.001&reactantsId=C00004&reactantsCoeff=2&reactantsName=NADH&reactantsPhase=aqueous&reactantsConcentration=0.001&reactantsId=C00008&reactantsCoeff=-1&reactantsName=ADP&reactantsPhase=aqueous&reactantsConcentration=0.001&reactantsId=C00009&reactantsCoeff=-1&reactantsName=Orthophosphate&reactantsPhase=aqueous&reactantsConcentration=0.001&reactantsId=C00010&reactantsCoeff=-1&reactantsName=CoA&reactantsPhase=aqueous&reactantsConcentration=0.001&reactantsId=C00011&reactantsCoeff=1&reactantsName=CO2&reactantsPhase=aqueous&reactantsConcentration=0.001&reactantsId=C00024&reactantsCoeff=1&reactantsName=Acetyl-CoA&reactantsPhase=aqueous&reactantsConcentration=0.001&reactantsId=C00067&reactantsCoeff=-3&reactantsName=Formaldehyde&reactantsPhase=aqueous&reactantsConcentration=0.001&reactantsId=C00001&reactantsCoeff=1&reactantsName=H2O&reactantsPhase=liquid&reactantsConcentration=1&ph=7.000000&pmg=14.000000&ionic_strength=0.100000&e_reduction_potential=0.000000&max_priority=0&mode=BA) | -110.2 | 1.9 | |
| **MCC pathway** | ΔrG'^m^ (kJ mol^-1^) | MDF(kJ mol^-1^) | |
| Formaldehyde + D-Ribulose-5-phosphate <=> D-arabino-6-Phospho-hex-3-ulose | -9.3 |  | |
| D-arabino-6-Phospho-hex-3-ulose <=> D-Fructose-6-phosphoric acid | -14.1 |  | |
| D-Fructose-6-phosphoric acid + D-Glyceraldehyde-3-phosphate <=> D-Xylulose-5-phosphate + D-Erythrose-4-phosphate | 10 |  | |
| D-Erythrose-4-phosphate + D-Fructose-6-phosphoric acid <=> D-Glyceraldehyde-3-phosphate + D-Sedoheptulose-7-phosphate | 0.7 |  | |
| D-Glyceraldehyde-3-phosphate + D-Sedoheptulose-7-phosphate <=> D-Ribose-5-phosphate + D-Xylulose-5-phosphate | 3.8 |  | |
| D-Xylulose-5-phosphate <=> D-Ribulose-5-phosphate | 3.4 |  | |
| D-Ribose-5-phosphate <=> D-Ribulose-5-phosphate | 1.9 |  | |
| Orthophosphate + D-Xylulose-5-phosphate <=> D-Glyceraldehyde-3-phosphate + Acetyl phosphate + H_2_O | -59.8 |  | |
| Acetyl phosphate + CoA<=> Acetyl-CoA + Phosphate | -9.8 |  | |
| **Overall reaction:** [CoA + 2 Formaldehyde <=> Acetyl-CoA + H_2_O](http://equilibrator.weizmann.ac.il/reaction?reactantsId=C00010&reactantsCoeff=-1&reactantsName=CoA&reactantsPhase=aqueous&reactantsConcentration=0.001&reactantsId=C00024&reactantsCoeff=1&reactantsName=Acetyl-CoA&reactantsPhase=aqueous&reactantsConcentration=0.001&reactantsId=C00067&reactantsCoeff=-2&reactantsName=Formaldehyde&reactantsPhase=aqueous&reactantsConcentration=0.001&reactantsId=C00001&reactantsCoeff=1&reactantsName=H2O&reactantsPhase=liquid&reactantsConcentration=1&ph=7.000000&pmg=14.000000&ionic_strength=0.100000&e_reduction_potential=0.000000&max_priority=0&mode=BA) | -96.7 | 5.8 | |

Note: The thermodynamic data come from the web interface of eQuilibrator (<http://equilibrator.weizmann.ac.il>)^32^

**Supplementary Table 2. Functional annotation of all the docking candidates.**

| **PDB ID** | Distance (Å) | Function annotation |
| --- | --- | --- |
| 4COK | 3.29 | Pyruvate decarboxylase |
| 2VBI | 3.37 | Pyruvate decarboxylase |
| 2WVG | 3.43 | Pyruvate decarboxylase |
| 2VK8 | 3.61 | Pyruvate decarboxylase |
| 4K9Q | 3.75 | Benzoylformate decarboxylase |
| 1R9J | 3.78 | Transketolase |
| 3FZN | 3.84 | Benzoylformate decarboxylase |
| 1OVM | 3.84 | Indolepyruvate decarboxylase |
| 2C42 | 3.85 | Pyruvate-ferredoxin oxidoreductase |
| 1TRK | 3.85 | Transketolase |
| 1ITZ | 3.88 | Transketolase |
| 4TKR | 4.01 | Thiamine transporter ThiT (protein) |
| 2JLC | 4.03 | Carboxylate synthase |
| 2UZ1 | 4.04 | Benzaldehyde lyase |
| 2VBF | 4.09 | Keto acid decarboxylase |
| 1OZH | 4.23 | Acetolactate synthase |
| 2IHT | 4.27 | Carboxyethylarginine synthase |
| 2PAN | 4.44 | Glyoxylate carboligase |
| 2X7J | 4.47 | Menaquinone biosynthesis protein |
| 4RJJ | 4.66 | Acetolactate synthase |
| 1Y9D | 5.00 | Pyruvate Oxidase |
| 5A65 | 5.36 | Thiamine triphosphatase |
| 2NXW | 5.41 | Phenylpyruvate decarboxylase |
| 2VK4 | 5.92 | Pyruvate decarboxylase |
| 2YIC | 6.14 | Alpha-ketoglutarate decarboxylase |
| 4FEG | 7.41 | Pyruvate oxidase |
| 3AI7 | 8.38 | Phosphoketolase |
| 2C31 | 8.70 | Oxalyl-COA decarboxylase |
| 3UPT | 10.16 | Transketolase |
| 2R5N | 10.43 | Transketolase |
| 3RIM | 10.49 | Transketolase |
| 3S4Y | 11.42 | Thiamin pyrophosphokinase |
| 2Q28 | 11.48 | Oxalyl-COA decarboxylase |
| 2PGN | 11.58 | Hydrolase |
| 2OZL | 11.89 | Pyruvate dehydrogenase |
| 5C4I | 11.94 | Oxalate Oxidoreductase |
| 1JSC | 13.05 | Acetohydroxyacid synthase |

**Supplementary Table 3. Data collection and refinement statistics of GALS.**

|  | **GALS** |
| --- | --- |
| **Data collection** |  |
| Space group | *C*121 |
| Cell dimensions |  |
| *a*, *b*, *c* (Å) | 104.9, 124.4, 97.8 |
| α, β, γ (°) | 90.0, 122.3, 90.0 |
| Resolution (Å) | 44.24-1.80(1.86-1.80) * |
| *R*_merge_(%) | 9.6(72.6) |
| *I* / σ*I* | 23.6(3.13) |
| Completeness (%) | 99.4(98.7) |
| Redundancy | 6.8(6.6) |
| **Refinement** |  |
| Resolution (Å) | 44.24-1.80 |
| No. reflections | 114,873 |
| *R*_work_ / *R*_free_ | 0.162/0.188 |
| No. atoms |  |
| Protein | 7,940 |
| ThDP | 52 |
| Mg^2+^ | 3 |
| Water | 1,001 |
| Average B factors (Å^2^) |  |
| Protein | 15.4 |
| ThDP | 16.6 |
| Mg^2+^ | 10.3 |
| Water | 26.4 |
| R.m.s. deviations |  |
| Bond lengths (Å) | 0.006 |
| Bond angles (°) | 0.87 |

*Values in parentheses are for highest-resolution shell.

**Supplementary Table 4. Plasmids used in this study.**

| **Plasmids** | **Relevant characteristics** | **Source** |
| --- | --- | --- |
| pET28a | pBR322 ori with *pT7*; *KanR* | Novagen |
| pET28a-*4COK* | pET28a vector, *Nde*І*-4COK-Xho*I | This study |
| pET28a-*4K9Q* | pET28a vector, *Nde*І*-4K9Q-Xho*I | This study |
| pET28a-*1R9J* | pET28a vector, *Nde*І*-1R9J-Xho*I | This study |
| pET28a-*3FZN* | pET28a vector, *Nde*І*-3FZN-Xho*I | This study |
| pET28a-*2JLC* | pET28a vector, *Nde*І*-2JLC-Xho*I | This study |
| pET28a-*2UZ1* | pET28a vector, *Nde*І*-2UZ1-Xho*I | This study |
| pET28a-*4FEG* | pET28a vector, *Nde*І*-4FEG-Xho*I | This study |
| pET28a-*2C31* | pET28a vector, *Nde*І*-2C31-Xho*I | This study |
| pET28a-*5C4I* | pET28a vector, *Nde*І*-5C4I-Xho*I | This study |
| pET28a-*GALS* | pET28a vector, *Nde*І-*BFD^W86R-N87T-L109G-L110E-A460M-H281V-Q282F^-Xho*I | This study |
| pET28a-*PK1* | pET28a vector, *Nde*І*-PK1-Xho*I | This study |
| pET28a-*PK2* | pET28a vector, *Nde*І*-PK2-Xho*I | This study |
| pET28a-*PK3* | pET28a vector, *Nde*І*-PK3-Xho*I | This study |
| pET28a-*PK4* | pET28a vector, *Nde*І*-PK4-Xho*I | This study |
| pET28a-*PK5* | pET28a vector, *Nde*І*-PK5-Xho*I | This study |
| pET28a-*PK6* | pET28a vector, *Nde*І*-PK6-Xho*I | This study |
| pET28a-*PK7* | pET28a vector, *Nde*І*-PK7-Xho*I | This study |
| pET28a-*PK8* | pET28a vector, *Nde*І*-PK8-Xho*I | This study |
| pET28a-*PTA* | pET28a vector, *Nde*І*-PTA-Xho*I | This study |
| pET28a-*GALS*-*ACPS*-*PTA* | pET28a vector, *Nco*І*-GALS- Nde*І, *BamH*I*-ACPS- Sac*І, *Not*І-*PTA*-*Xho*I | This study |
| pET28a-*GALS* -*PTA* | pET28a vector, *Nco*І*-GALS- Nde*І, *Not*І-*PTA*-*Xho*I | This study |
| pET28a-*GALS*-*ACPS* | pET28a vector, *Nco*І*-GALS- Nde*І, *BamH*I*-ACPS- Sac*І, | This study |
| pCDFDuet-1 | CloDF13 ori, T7, *SmR* | This study |
| *BsMDH*-pCDF | pCDFDuet-1 vector, *Nco*І*-BsMDH- Not*І | This study |

**References**

1. Jordan, F. Current mechanistic understanding of thiamin diphosphate-dependent enzymatic reactions. *Nat Prod Rep* **20**, 184-201 (2003).

2. Li, W. & Godzik, A. Cd-hit: a fast program for clustering and comparing large sets of protein or nucleotide sequences. *Bioinformatics* **22**, 1658-1659 (2006).

3. Lang, P.T., Brozell, S.R., Mukherjee, S., Pettersen, E.F., Meng, E.C. *et al.* DOCK 6: combining techniques to model RNA-small molecule complexes. *Rna* **15**, 1219-1230 (2009).

4. Pettersen, E.F., Goddard, T.D., Huang, C.C., Couch, G.S., Greenblatt, D.M. *et al.* UCSF Chimera--a visualization system for exploratory research and analysis. *J Comput Chem* **25**, 1605-1612 (2004).

5. Gasteiger, J. & Marsili, M. Iterative partial equalization of orbital electronegativity—a rapid access to atomic charges. *Tetrahedron* **36**, 3219-3228 (1980).

6. Wang, J., Wang, W., Kollman, P.A. & Case, D.A. Automatic atom type and bond type perception in molecular mechanical calculations. *J Mol Graph Model* **25**, 247-260 (2006).

7. Poust, S., Piety, J., Bar-Even, A., Louw, C., Baker, D. *et al.* Mechanistic analysis of an engineered enzyme that catalyzes the formose reaction. *Chembiochem* **16**, 1950-1954 (2015).

8. Yupeng Liu, H.L., Yang Sun, Zhongsai Zhou. Determination of 1,3-dihydroxyacetone in fermentation broth by spectrophotometry. *Chinese Journal of Pharmaceuticals* **42**, 834-837 (2011).

9. Reetz, M.T. & Carballeira, J.D. Iterative saturation mutagenesis (ISM) for rapid directed evolution of functional enzymes. *Nat Protoc* **2**, 891-903 (2007).

10. Otwinowski, Z. & Minor, W. Processing of X-ray diffraction data collected in oscillation mode. *Methods Enzymol* **276**, 307-326 (1997).

11. Adams, P.D., Afonine, P.V., Bunkoczi, G., Chen, V.B., Davis, I.W. *et al.* PHENIX: a comprehensive Python-based system for macromolecular structure solution. *Acta Crystallogr D Biol Crystallogr* **66**, 213-221 (2010).

12. Hasson, M.S., Muscate, A., McLeish, M.J., Polovnikova, L.S., Gerlt, J.A. *et al.* The crystal structure of benzoylformate decarboxylase at 1.6 Å resolution: diversity of catalytic residues in thiamin diphosphate-dependent enzymes. *Biochemistry* **37**, 9918-9930 (1998).

13. Murshudov, G.N., Skubak, P., Lebedev, A.A., Pannu, N.S., Steiner, R.A. *et al.* REFMAC5 for the refinement of macromolecular crystal structures. *Acta Crystallogr D Biol Crystallogr* **67**, 355-367 (2011).

14. Emsley, P. & Cowtan, K. Coot: model-building tools for molecular graphics. *Acta Crystallogr D Biol Crystallogr* **60**, 2126-2132 (2004).

15. Laskowski, R.A., Macarthur, M. W., Moss, D. S. & Thornton, J. M. Procheck - a program to check the stereochemical quality of protein structures. *J Appl Crystallogr* **26**, 283-291 (1993).

16. Winn, M.D., Ballard, C.C., Cowtan, K.D., Dodson, E.J., Emsley, P. *et al.* Overview of the CCP4 suite and current developments. *Acta Crystallogr D Biol Crystallogr* **67**, 235-242 (2011).

17. Krissinel, E. & Henrick, K. Inference of macromolecular assemblies from crystalline state. *J Mol Biol* **372**, 774-797 (2007).

18. Ollikainen, N., de Jong, R.M. & Kortemme, T. Coupling protein side-chain and backbone flexibility improves the re-design of protein-ligand specificity. *PLoS Comput Biol* **11** (2015).

19. Durrant, J.D., de Oliveira, C.A. & McCammon, J.A. POVME: an algorithm for measuring binding-pocket volumes. *J Mol Graph Model* **29**, 773-776 (2011).

20. Davis, I.W. & Baker, D. RosettaLigand docking with full ligand and receptor flexibility. *J Mol Biol* **385**, 381-392 (2009).

21. O'Boyle, N.M., Banck, M., James, C.A., Morley, C., Vandermeersch, T. *et al.* Open Babel: An open chemical toolbox. *J Cheminform* **3**, 33 (2011).

22. Biasini, M., Bienert, S., Waterhouse, A., Arnold, K., Studer, G. *et al.* SWISS-MODEL: modelling protein tertiary and quaternary structure using evolutionary information. *Nucleic Acids Res* **42**, 252-258 (2014).

23. Kiefer, F., Arnold, K., Kunzli, M., Bordoli, L. & Schwede, T. The SWISS-MODEL repository and associated resources. *Nucleic Acids Res* **37**, D387-392 (2009).

24. Arnold, K., Bordoli, L., Kopp, J. & Schwede, T. The SWISS-MODEL workspace: a web-based environment for protein structure homology modelling. *Bioinformatics* **22**, 195-201 (2006).

25. Guex, N., Peitsch, M.C. & Schwede, T. Automated comparative protein structure modeling with SWISS-MODEL and SWISS-PDBVIEWER: a historical perspective. *Electrophoresis* **30**, 162-173 (2009).

26. DeLano, W. PyMOL. (2002).

27. Zhang, J. & Liu, Y. Computational studies on the catalytic mechanism of phosphoketolase. *Comput. Theor. Chem.* **1025**, 1-7 (2013).

28. M.J. Frisch et al. Gaussian 03, revision C. 02. (2008).

29. Siegel, J.B., Smith, A.L., Poust, S., Wargacki, A.J., Bar-Even, A. *et al.* Computational protein design enables a novel one-carbon assimilation pathway. *Proc Natl Acad Sci USA* **112**, 3704-3709 (2015).

30. Bruning, M., Berheide, M., Meyer, D., Golbik, R., Bartunik, H. *et al.* Structural and kinetic studies on native intermediates and an intermediate analogue in benzoylformate decarboxylase reveal a least motion mechanism with an unprecedented short-lived predecarboxylation intermediate. *Biochemistry* **48**, 3258-3268 (2009).

31. Yuan, Y. et al. Overexpression of the Lactobacillus plantarum peptidoglycan biosynthesis murA2 gene increases the tolerance of Escherichia coli to alcohols and enhances ethanol production. *Appl Microbiol Biotechnol* **98**, 8399-8411 (2014).

32. Flamholz, A., Noor, E., Bar-Even, A. & Milo, R. eQuilibrator--the biochemical thermodynamics calculator. *Nucleic Acids Res* **40**, D770-775 (2012).
