# Supplementary material for: Constructing a synthetic pathway for acetyl-coenzyme A from one-carbon through enzyme design
Source: Nat Commun. 2019 Mar 26;10:1378. doi: 10.1038/s41467-019-09095-z (PMC6435721; doi:10.1038/s41467-019-09095-z)
Supplement: Supplementary file 3 — Additional Supplementary Files [file 41467_2019_9095_MOESM3_ESM.pdf]

Title: Supplementary Data 1:
Description: The backrub option file and docking parameters

Title: Supplementary Data 2:
Description: POVME configuration file

Title: Supplementary Data 3:
Description: The primers and DNA sequences used in this study.
