# Supplementary material for: Constructing a synthetic pathway for acetyl-coenzyme A from one-carbon through enzyme design
Source: Nat Commun. 2019 Mar 26;10:1378. doi: 10.1038/s41467-019-09095-z (PMC6435721; doi:10.1038/s41467-019-09095-z)
Supplement: Supplementary file 4 — Supplementary Data 1 [file 41467_2019_9095_MOESM4_ESM.docx]

**Backrub option file:**

-database /home/user/Programs/rosetta_2015/main/database

-s 1BFD_AB_clean_AL403F_Tem.pdb

-extra_res_fa ThDP_A.params ThDP_B.params

-ignore_unrecognized_res

-ex1

-ex2

-nstruct 500

-backrub:ntrials 1000

-resfile 1BFD_AB_clean_AL403F.resfile

-backrub::sc_prob_uniform 0.05

-out:file:scorefile score_backrub_AL403F.sc

-out:level 100

#-out:nooutput

**The Rosetta options to dock IMA:**

-in:file:s inputs_IMA/GALS_IMA.pdb

-in:file:extra_res_fa inputs_IMA/IMA.params

-packing

-ex1

-ex2aro

-ex2

-no_optH false

-flip_HNQ true

-ignore_ligand_chi true

-parser

-protocol inputs_IMA/ligand_dock.xml

-out

-path:all outputs_IMA

-nstruct 10000

-overwrite

**The Rosetta scripts to dock IMA:**

This protocol will simply do high-resolution docking.

It will also report the binding energy (ddg) and buried-surface area (sasa) in the score file.

<ROSETTASCRIPTS>

<SCOREFXNS>

<ligand_soft_rep weights="ligand_soft_rep">

</ligand_soft_rep>

<hard_rep weights="ligand">

</hard_rep>

</SCOREFXNS>

<LIGAND_AREAS>

<docking_sidechain_X chain="X" cutoff="6.0" add_nbr_radius="true" all_atom_mode="true" minimize_ligand="10"/>

<final_sidechain_X chain="X" cutoff="6.0" add_nbr_radius="true" all_atom_mode="true"/>

<final_backbone_X chain="X" cutoff="7.0" add_nbr_radius="false" all_atom_mode="true" Calpha_restraints="0.3"/>

</LIGAND_AREAS>

<INTERFACE_BUILDERS>

<side_chain_for_docking ligand_areas="docking_sidechain_X"/>

<side_chain_for_final ligand_areas="final_sidechain_X"/>

<backbone ligand_areas="final_backbone_X" extension_window="3"/>

</INTERFACE_BUILDERS>

<MOVEMAP_BUILDERS>

<docking sc_interface="side_chain_for_docking" minimize_water="true"/>

<final sc_interface="side_chain_for_final" bb_interface="backbone" minimize_water="true"/>

</MOVEMAP_BUILDERS>

<SCORINGGRIDS ligand_chain="X" width="15">

<classic grid_type="ClassicGrid" weight="1.0"/>

</SCORINGGRIDS>

<MOVERS>

<Transform name="transform" chain="X"box_size="7.0" move_distance="0.2" angle="20" cycles="500" repeats="1" temperature="5"/>

<HighResDocker name="high_res_docker" cycles="6" repack_every_Nth="3" scorefxn="ligand_soft_rep" movemap_builder="docking"/>

<FinalMinimizer name="final" scorefxn="hard_rep" movemap_builder="final"/>

<InterfaceScoreCalculator name="add_scores" chains="X" scorefxn="hard_rep"/>

</MOVERS>

<PROTOCOLS>

<Add mover_name="transform"/>

<Add mover_name="high_res_docker"/>

<Add mover_name="final"/>

<Add mover_name="add_scores"/>

</PROTOCOLS>

</ROSETTASCRIPTS>
