# Supplementary material for: Constructing a synthetic pathway for acetyl-coenzyme A from one-carbon through enzyme design
Source: Nat Commun. 2019 Mar 26;10:1378. doi: 10.1038/s41467-019-09095-z (PMC6435721; doi:10.1038/s41467-019-09095-z)
Supplement: Supplementary file 5 — Supplementary Data 2 [file 41467_2019_9095_MOESM5_ESM.docx]

# POVME 2.0 Sample Input File

# First, we need to define a point field that entirely encompasses all trajectory pockets.

GridSpacing 0.50 # The distance, in Angstroms, between

# adjacent points. Making this number

# lower improves accuracy at the

# expense of compute time.

PointsInclusionSphere 109.20 44.00 90.70 6.0 # Add a sphere of points to the pocket-

# encompassing region, centered on

# (65.0 98.0 50.0) with radius 16.0.

# (65.0 98.0 50.0) is the location of

# the enzymatic active site in the

# sample PDB trajectory file provided

# (4NSS.pdb).

#PointsInclusionSphere -100.0 -100.0 -100.0 10.0 # Add a second sphere of points. Note

# that this sphere is included only for

# demonstration purposes. The point

# (-100.0 -100.0 -100.0) is actually far

# from the 4NSS.pdb structure.

#PointsInclusionBox 100.0 100.0 100.0 10.0 10.0 10.0 # Add a rectangular prism ("box") of

# points to the pocket-encompassing

# region, centered on (100.0 100.0 100.0)

# and spanning 10.0 Angstroms in the x,

# y, and z directions, respectively.

# Again, this box is far from the

# 4NSS.pdb structure and is included only

# for demonstration purposes.

#PointsExclusionSphere 65.0 98.0 60.0 5.0 # Remove all points from the pocket-

# encompassing region that fall within

# a sphere centered at

# (-100.0 -100.0 -100.0) with radius

# 10.0.

#PointsExclusionBox 100.0 100.0 100.0 10.0 10.0 10.0 # Remove all points from the pocket-

# encompassing region that fall within a

# box, centered at (100.0 100.0 100.0)

# and spanning 10.0 Angstroms in the x,

# y, and z directions, respectively.

# Saving and loading the POVME points of the pocket-encompassing region.

SavePoints true # You can optionally save the point

# field to a PDB file. As you can

# imagine, identifying just the right set

# of inclusion and exclusion spheres and

# boxes to encompass the binding pocket

# is challenging. One approach is to

# define an initial geometry, visualize

# that geometry together with the protein

# using a program like VMD, and then

# iteratively add new inclusion and

# exclusion regions as required. The

# ability to save the points for

# visualization is helpful. Additionally,

# if your point field contains many

# points, generating the field may be

# computationally intensive. If you want

# to use the same field for multiple

# POVME runs, using a saved copy of the

# field rather than repeatedly generating

# it from scratch is more efficient. Note

# that POVME also saves a NPY file, which

# contains the same data as the PDB file

# but can be loaded much faster in

# subsequent POVME runs. The point-field

# PDB file is saved to

# {PREFIX}point_field.pdb (see

# OutputFilenamePrefix below).

# Additionally, if you specify a

# contiguous-pocket seed region (see

# ContiguousPocketSeedSphere and

# ContiguousPocketSeedBox below), POVME

# will also save those points to

# {PREFIX}contiguous_pocket_seed_points.pdb

# for visualization.

# Tell POVME how to save the output

OutputFilenamePrefix ./POVME_round1/POVME_ # All the files POVME outputs will start

# with this prefix. POVME automatically

# creates any required directory

# (./POVME_test_run/ in this case).

#LoadPointsFilename points.pdb.npy # You can optionally load previous point

# fields if you don't want to generate

# them using the comamnds above. Note

# that you should use the .pdb.npy file,

# not the pdb file.

# Load the PDB trajectory file

PDBFileName 3FZN_TPP_round1.pdb # Load in the PDB trajectory file with

# the pocket you want to analyze.

# Tell POVME how to identify points that are within the binding pocket.

DistanceCutoff 1.00 # Any point that comes within this

# distance of any receptor atom's van der

# Waals surface will not be considered

# part of the pocket volume. 1.09

# Angstroms was chosen as the default

# value because that is the van der Waals

# radius of a hydrogen atom.

ConvexHullExclusion true # Calculate the convex hull of receptor

# atoms near the pocket. Remove portions

# of the binding pocket that fall outside

# this convex hull. Setting this to false

# will speed up the calculation but may

# lead to inaccuracies in some cases.

ContiguousPocketSeedSphere 108.00 43.00 91.50 2.0 # It's possible your pocket-encompassing

# point field defined above might include

# more than one pocket in at least some

# of the frames of your trajectory. You

# can instruct POVME to remove any points

# that are not contiguous with a user-

# defined "contiguous pocket seed

# region." This region, which is typically

# just a small sphere placed in the

# center of your primary pocket of

# interest, tells POVME which pocket to

# measure. If no such regions are

# specified, POVME will calculate the

# total volume accross all pockets

# covered by your pocket-encompassing

# point field, even if they are not

# contiguous.

#ContiguousPocketSeedBox 50.0 50.0 50.0 10.0 10.0 10.0 # You can add additional volumes to the

# contiguous pocket seed region with

# additional ContiguousPocketSeedSphere

# and ContiguousPocketSeedBox commands,

# following the notation described above.

ContiguousPointsCriteria 6 # Two pocket volumes are considered

# "contiguous" if they share at least

# this number neighboring points in

# common. Note that points that are

# "kitty-corner" from each other count

# as neighbors.

# Tell POVME how to perform the calculations.

NumProcessors 8 # POVME can use multiple processors on

# Unix-based systems.

UseDiskNotMemory true # In some cases, your PDB trajectory may

# be so large that the resulting POVME

# analysis cannot be easily stored in

# your computer's memory. If

# UseDiskNotMemory is set to true, POVME

# will rely more on your disk space than

# on memory/RAM.

SaveIndividualPocketVolumes true # If true, POVME saves the pocket-volume

# points of each frame to a separate PDB

# file. The file names will be like

# {PREFIX}frame_X.pdb.

SavePocketVolumesTrajectory false # If true, POVME saves all the pocket-

# volume points of each frame to a single

# PDB trajectory file. The individual

# frames are separated by END cards. The

# file name will be

# {PREFIX}volume_trajectory.pdb.

OutputEqualNumPointsPerFrame true # Some visualization programs (e.g. VMD)

# are only compatible with trajectories

# that have the same number of atoms in

# each frame. If EqualNumAtomsPerFrame is

# true, POVME adds extra points at the

# origin (0.0, 0.0, 0.0) to satisfy this

# requirement. This affects files created

# with both SaveIndividualPocketVolumes

# and SavePocketVolumesTrajectory but

# does not alter the volume calculation

# itself.

SaveTabbedVolumeFile true # If true, POVME saves the calculated

# volumes to a file in a simple tabular

# format that can be easily pasted into

# popular spreadsheet programs like

# Microsoft Excel. The file is named

# {PREFIX}volumes.tabbed.txt

SaveVolumetricDensityMap false # If true, POVME saves a volumetric

# density map in the DX format. A

# volumetric density value is associated

# with each of the pocket-occupying

# points by calculating the fraction of

# all trajectory pocket volumes that

# include the given point. The file is

# named {PREFIX}volumetric_density.dx.

CompressOutput false # If you're short on disk space, POVME

# can automatically compress all output

# files using gz compression.
